# The Draft Genome of the Endangered Sichuan Partridge (*Arborophila rufipectus*) with Evolutionary Implications

**DOI:** 10.3390/genes10090677

**Published:** 2019-09-05

**Authors:** Chuang Zhou, Hongmei Tu, Haoran Yu, Shuai Zheng, Bo Dai, Megan Price, Yongjie Wu, Nan Yang, Bisong Yue, Yang Meng

**Affiliations:** 1 Key Laboratory of Bioresources and Ecoenvironment (Ministry of Education), College of Life Sciences, Sichuan University, Chengdu 610064, China (C.Z.) (H.T.) (H.Y.) (S.Z.) (M.P.) (Y.W.) (B.Y.); 2 College of Life Sciences, Leshan Normal University, Leshan 614004, China; 3 Institute of Qinghai-Tibetan Plateau, Southwest Minzu University, Chengdu 610064, China

**Keywords:** Sichuan partridge, comparative genomics, olfactory receptor, positive selection, demography

## Abstract

The Sichuan partridge (*Arborophila rufipectus*, Phasianidae, Galliformes) is distributed in south-west China, and classified as endangered grade. To examine the evolution and genomic features of Sichuan partridge, we de novo assembled the Sichuan partridge reference genome. The final draft assembly consisted of approximately 1.09 Gb, and had a scaffold N50 of 4.57 Mb. About 1.94 million heterozygous single-nucleotide polymorphisms (SNPs) were detected, 17,519 protein-coding genes were predicted, and 9.29% of the genome was identified as repetitive elements. A total of 56 olfactory receptor (OR) genes were found in Sichuan partridge, and conserved motifs were detected. Comparisons between the Sichuan partridge genome and chicken genome revealed a conserved genome structure, and phylogenetic analysis demonstrated that *Arborophila* possessed a basal phylogenetic position within Phasianidae. Gene Ontology (GO) enrichment analysis of positively selected genes (PSGs) in Sichuan partridge showed over-represented GO functions related to environmental adaptation, such as energy metabolism and behavior. Pairwise sequentially Markovian coalescent analysis revealed the recent demographic trajectory for the Sichuan partridge. Our data and findings provide valuable genomic resources not only for studying the evolutionary adaptation, but also for facilitating the long-term conservation and genetic diversity for this endangered species.

## 1. Introduction

**Figure 1:**
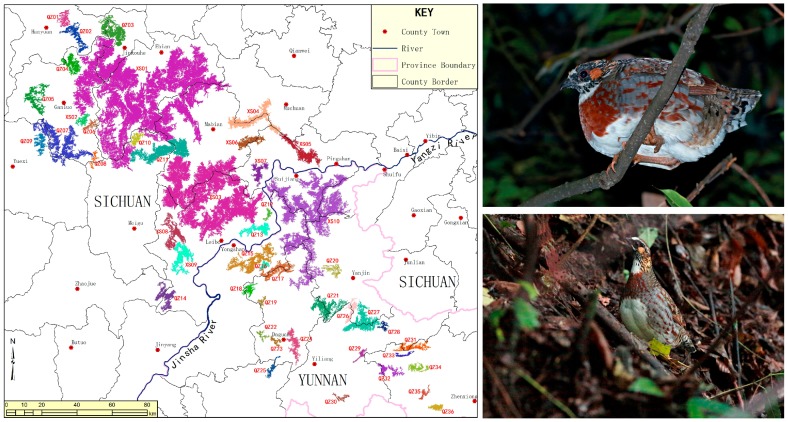
Distribution and photos of the Sichuan partridge.

The Sichuan partridge (*Arborophila rufipectus*, Phasianidae, and Galliformes) is endemic to the mountains in the south-west China [1]. It has been classified as an endangered species (IUCN 2007) and a nationally protected animal in China because of its largely restricted range, very small population size, and severely fragmented habitat (Figure 1) [2,3]. Owing to the habitat fragmentation of endemic birds in subtropical forest in the mountains of Southwestern China, the populations of Sichuan partridge have decreased dramatically [4], which has led to governmental protection of the Sichuan partridge in much of their range. To date, few studies have been conducted for the investigation associated with the genetic mechanisms of the environmental adaption of the Sichuan partridge, and the whole genome of Sichuan partridge is currently not available. Further research is essential to clarify the environmental adaption and evolutionary history of Sichuan partridge and to elucidate functional genomic regions that underlie ecological adaptation. To help achieve the goal, we have sequenced and assembled the Sichuan partridge reference genome, and performed comparative genomics analysis. The whole genome of Sichuan partridge will be a valuable genomic resource for answering evolutionary questions associated with this endangered species, and for the development of genetic tools for Sichuan partridge research and conservation.

## 2. Materials and Methods

### 2.1. Sequencing, Assembly, and Genome Data

Muscle tissue was collected from a wild male Sichuan partridge which was preserved in the Natural History Museum of Sichuan University. Collected muscle tissue was used for genomic DNA extraction, isolation, and sequencing. Three paired-end libraries with insert sizes of 250 base pairs (bp), 500 bp, and 800 bp, and five mate-paired libraries with insert sizes of 2 kb, 5 kb, 10 kb, 15 kb, and 20 kb were constructed. The libraries were sequenced on an Illumina 2000 platform (Illumina, San Diego, CA, USA).

Before assembly, 17-Kmer analysis was performed for the genome size estimation of the Sichuan partridge, and the assembly was first analyzed by SOAPdenovo2 [5]. After using SSPACE [6] to build the super-scaffolds, intra-scaffold gaps were then filled using Gapcloser with reads from short-insert libraries. CEGMA (v2.5) [7] and BUSCO (v3.1.0) [8] were employed to evaluate the genome completeness. Gene prediction and functional annotation were conducted according to our previous study [9]. The raw data is available as a Bioproject at NCBI (PRJNA419836).

To conduct comparative genomic analysis, we collected complete genomes of seven other birds from NCBI (turkey, *Meleagris gallopavo*, GCF_000146605.2; chicken, *Gallus gallus*, GCF_000002315.6; mallard, *Anas platyrhynchos*, GCF_003850225.1; ostrich, *Struthio camelus*, GCF_000698965.1; peregrine falcon, *Falco peregrinus*, GCF_000337955.1; zebra finch, *Taeniopygia guttata*, GCF_000151805.1; rock pigeon, *Columba livia*, GCF_000337935.1). The complete genome of Hainan partridge (*Arborophila ardens*) and Chinese monal (*Lophophorus lhuysii*) were sequenced and assembled by our lab [9,10].

### 2.2. Characterization of Repeat Content

Repetitive elements in Sichuan partridge, Hainan partridge, Chinese monal, turkey, and chicken were identified using RepeatMasker (http://www.repeatmasker.org/). Perl script “calcDivergence-fromAlign.pl” in the RepeatMasker package was used to calculate the Kimura 2-parameter [11] distance between a transposable element insertion and the assumed ancestral sequence. Microsatellites (SSRs) in the genome of the above five Phasianidae species were identified by the software Krait [12] with default settings.

### 2.3. Analyses of Olfactory Receptor (ORs) Genes

For the identification of OR genes in birds, we employed a consensus approach, because it was reported that different approaches and/or cutoffs would possibly cause systematic bias [13]. A homology search was conducted to identify OR genes in these ten avian genomes. The method to detect functional OR genes in the ten bird genome sequences was similar to our previous studies [13,14], but we improved it to be applicable to any bird species. We aligned a total of 477 functional OR amino acid sequences obtained from the ten avian species using MAFFT 7 with auto strategy parameters. An unrooted phylogenetic tree was constructed after 1000 rounds of bootstrapping using IQ-TREE (v1.6.10) [15] with maximum likelihood (ML) approaches on the basis of the best-fit model JTT+F+R8 of amino acid evolution. We classified the identified OR genes into families and subfamilies based on the results of phylogenetic analyses and clustering analysis, and the multispecies OR gene clustering analysis was performed using CD-HIT software (v4.8.1) [16] (cutoff value of 40% and 60% amino acid similarity were set as the thresholds to discriminate between families and subfamilies, respectively) as previously described [17]. Sequence logos were generated from the alignment of the functional OR protein sequences for the identification of the conserved motifs in the predicted OR amino acid sequences with the program Multiple Expectation Maximization for Motif Elicitation (MEME) [18]. Only the top five conserved motifs were detected, with the motif length ranging from 5 to 50.

### 2.4. Gene Family and Positive Selection

The software orthoMCL (v2.0.9) [19] was used to define the orthologous genes from ten studied avian genomes (Sichuan partridge, Hainan partridge, Chinese monal, turkey, chicken, mallard, peregrine falcon, zebra finch, ostrich, and rock pigeon). Based on 1:1 orthologous genes aligned by PRANK (v.170427) [20] and concatenated to one sequence for each species, we constructed the phylogenetic tree using RAxML (v8.2.12) [21] with 1000 bootstrap replicates. The phylip dataset has been deposited at Figshare under the link https://figshare.com/articles/Sichuan_partridge/9751982. Divergence time estimation was performed by PAML MCMCTREE (v4.4) [22] as described in previous studies [23,24]. We used two calibrations for this analysis: (1) Divergence of the chicken and zebra finch was set to an age of 58.2–92.2 million years ago [10]; and (2) divergence of the chicken and duck was set to an age of 53.6–72 million years ago [23]. The alignments of 1:1 orthologous genes and phylogenetic tree were employed to estimate the ratio of the rates of non-synonymous-to-synonymous substitutions per gene (ω) by ML with the “codeml” program from PAML package [22] under the branch-site model. Two models were conducted to test the statistical significance of selective pressure specifically on the Sichuan partridge branch. One was the one-ratio model acting as the null model (NSsites = 0, model = 0), and the other was model 2 (NSsites = 2). The two models were compared with the likelihood ratio test (LRT), which was calculated from the log likelihood (lnL) values for both models. The *p*-values were obtained by calculating twice the difference between lnL (model2) and lnL (one-ratio) and compared with a χ-square distribution. We then identified positively selected genes (PSGs) of the Sichuan partridge by means of FDR adjustment with *Q*-values < 0.05. Gene Ontology (GO) enrichment of the PSGs of Sichuan partridge was performed via KOBAS 3.0 [25,26].

### 2.5. Demography Reconstruction

The software SAMtools was employed to detect single-nucleotide polymorphisms (SNPs) between diploid chromosomes for the Sichuan partridge [27]. Then we used the pairwise sequentially Markovian coalescent (PSMC) to infer the demographic history of the Sichuan partridge [28].

## 3. Results

### 3.1. Genome Sequencing, Assembly, and Quality Assessment

A total of 296.74 Gb (~272-fold coverage) of high-quality reads were generated for the Sichuan partridge after filtering out low quality and duplicated reads (Table 1). Based on the *K*-mer analysis, the genome size of Sichuan partridge was estimated to be 1.09 Gb, which was similar to other reported avian genomes. The final Sichuan partridge genome assembly consisted of approximately 1.09 Gb, with the contig N50 and scaffold N50 of 0.93 Kb and 4.57 Mb, respectively. CEGMA revealed 77.42% complete and 85.89% partial gene set for the assembled Sichuan partridge genome (Table S1), while BUSCO indicated the presence of 86.5% of the eukaryotic single-copy genes (Table S2).

**Figure 2:**
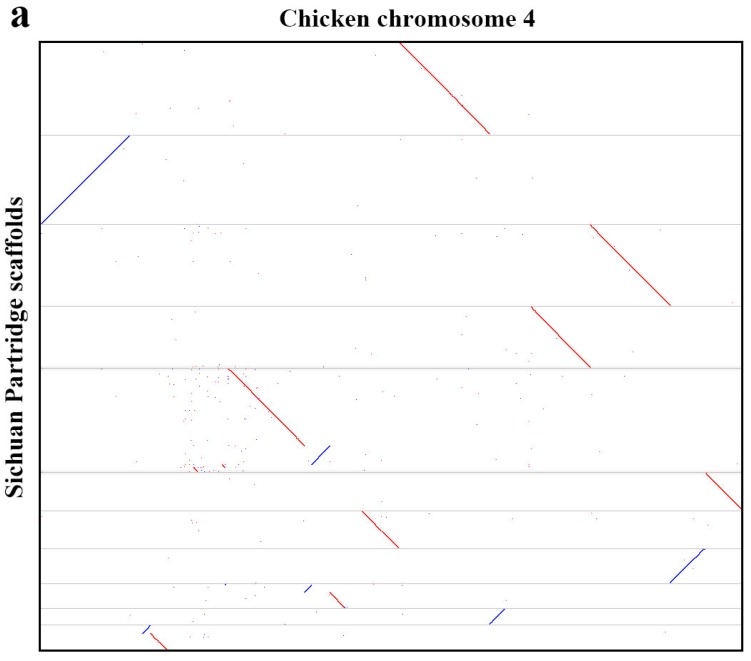

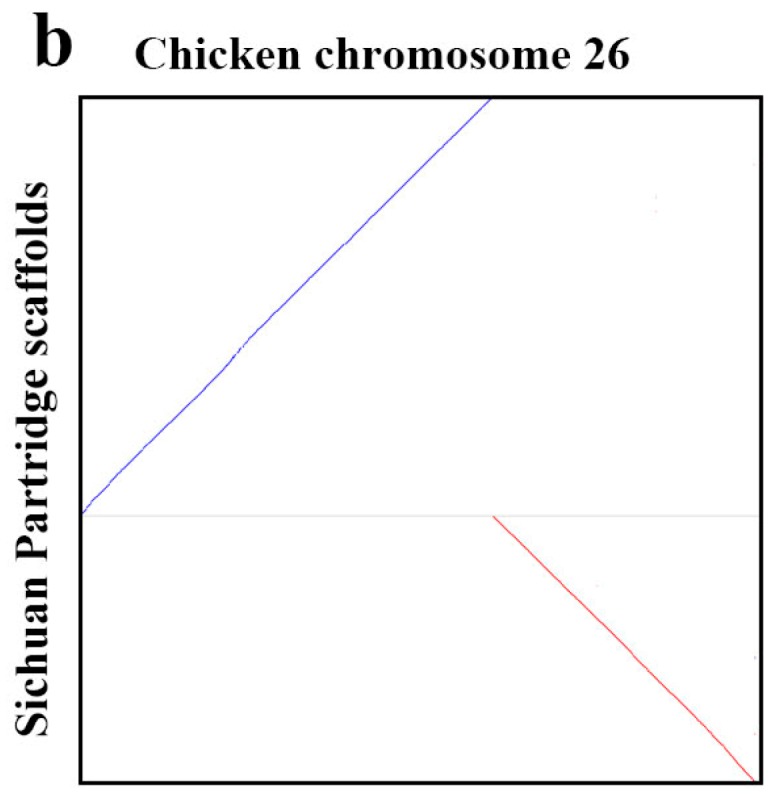
Alignments of Sichuan partridge scaffolds to chicken chromosomes. (**a**) Several chromosomal rearrangements between chicken and Sichuan partridge are evident in the chicken chromosome 4, while (**b**) the alignment of Sichuan Partridge scaffolds to chicken chromosome 26 is an example of scaffolds approaching chromosome size, high synteny and high assembly correctness. Forward alignments are in blue, reverse alignments are in red.

The high synteny and assembly correctness were illustrated through the alignment of Sichuan partridge scaffolds to the chicken reference genome (Figure S1). Inversions and chromosomal rearrangements were evident like chromosome 4 of the chicken (Figure 2a), and some of the chicken microchromosomes such as chromosome 26 can be covered almost entirely by a small number of Sichuan partridge scaffolds (Figure 2b).

### 3.2. Genome Characterization

**Figure 3:**
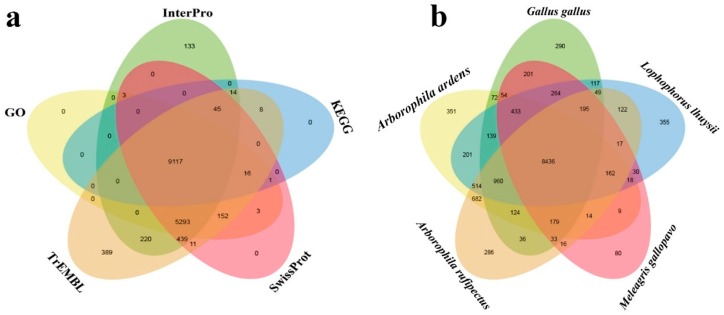

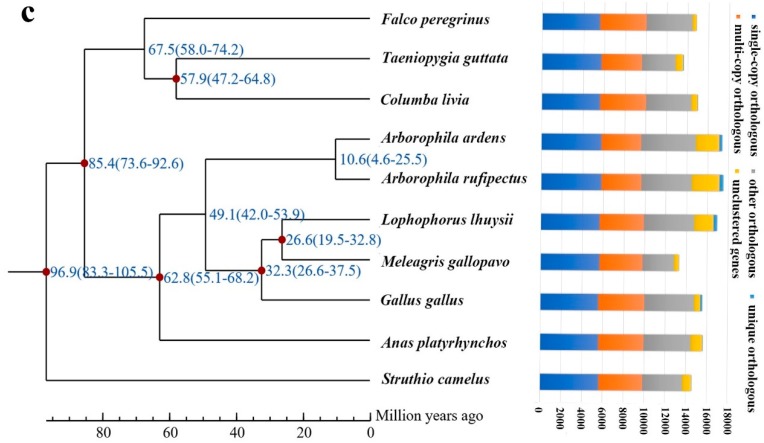
Comparative genomics in avian species studied. (**a**) Functional annotation of Sichuan partridge genes. (**b**) Orthologous gene clusters in five Phasianidae species. (**c**) Phylogenetic tree constructed using 1:1 orthologous genes. The time lines indicate divergence times among the species.

The Sichuan partridge genome had a GC content of approximately 41.95%, which was similar to other bird species such as chicken and zebra finch. Through gene prediction, we obtained a total of 17,519 protein-coding genes (PCGs) for the Sichuan partridge, of which 15,891 (90.71%) were well supported by public protein databases: TrEMBL (https://www.uniprot.org/statistics/TrEMBL), Swissprot (https://www.uniprot.org/), Interpro (https://www.ebi.ac.uk/interpro/), Nr (https://www.ncbi.nlm.nih.gov/refseq/about/nonredundantproteins/), GO (http://geneontology.org/), and KEGG (https://www.genome.jp/kegg/) (Figure 3a and Table S3).

**Figure 4:**
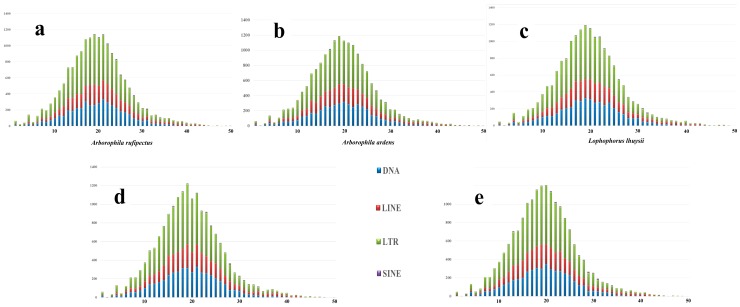
Divergence rate distribution of four major types of transposable elements (TEs) in (**a**) Sichuan partridge (*Arborophila rufipectus*), (**b**) Hainan partridge (*Arborophila ardens*), (**c**) Chinese monal (*Lophophorus lhuysii*), (**d**) turkey (*Meleagris gallopavo*), and (**e**) chicken (*Gallus gallus*) genomes. The divergence rate was calculated between their identified TE elements in the genome and the consensus sequences (Repbase).

About 101.22 Mb sequences (approximately 9.29% of the genome assembly) were attributed to repeats in Sichuan partridge genome. The percentage of long interspersed nuclear elements (LINEs), long terminal repeats (LTRs), short interspersed nuclear elements (SINEs), and DNA transposons were 6.35%, 1.77%, 0.05%, and 1.05% in the Sichuan partridge genome (Table 2). The divergence rate distribution of four major types of transposable elements in Sichuan partridge, Hainan partridge, Chinese monal, turkey, and chicken is shown in Figure 4. The repeat estimation of Sichuan partridge was larger than that of Hainan partridge, while smaller than those of Chinese monal, turkey, and chicken. However, read-based scaffolding which was involved in the insertion of “N’s” into gaps was demonstrated to lead to the underestimation of genome-wide repetitive content [29]. Even so, there is a common feature of the Sichuan partridge, turkey, scarlet macaw, zebra finch, and northern bobwhite genomes: the high proportion of L3/CR1 interspersed repeats [29,30,31,32] which are conserved across these divergent avian lineages.

For further evaluation of the repetitive content of the Sichuan partridge genome, we employed Krait to predict and characterize genome-wide SSR loci, which can identify the loci that could be used for population genetic studies. Imperfect SSRs were the most frequent type, followed by the perfect SSRs, and the least was the compound SSRs in the Sichuan partridge genome (Table S4). In total, we identified 367,513 perfect SSR loci containing 1 to 6 bp sequence motifs (Table 3). The total number of perfect SSR loci in the Sichuan partridge was larger than that in turkey and Chinese monal, while lower than that in Hainan partridge and chicken. The most frequent perfect SSRs were mononucleotide SSRs, with the highest frequencies of 256.49 loci/Mb and the highest densities of 4619.86 bp/Mb, accounting for 71.58% of the total number of SSRs of the Sichuan partridge genome. The second most frequent SSRs were tetranucleotide SSRs with a proportion of 11.49% for the Sichuan partridge. In contrast, dinucleotide, trinucleotide, and pentanucleotide SSRs were less frequent, and the least was hexanucleotide SSRs, only accounted for 0.58% of all of the SSRs for the Sichuan partridge. The most abundant motif categories found in the Sichuan partridge genome was revealed in Table S5. Importantly, microsatellite genotyping can be utilized to assess the population structure, gene flow, and covey composition within and between Sichuan partridge populations, and thus, the resources described herein can be used for development of genetic markers for the Sichuan partridge.

### 3.3. Olfactory Receptors (ORs): Composition, Classification, and Conserved Motifs

**Figure 5:**
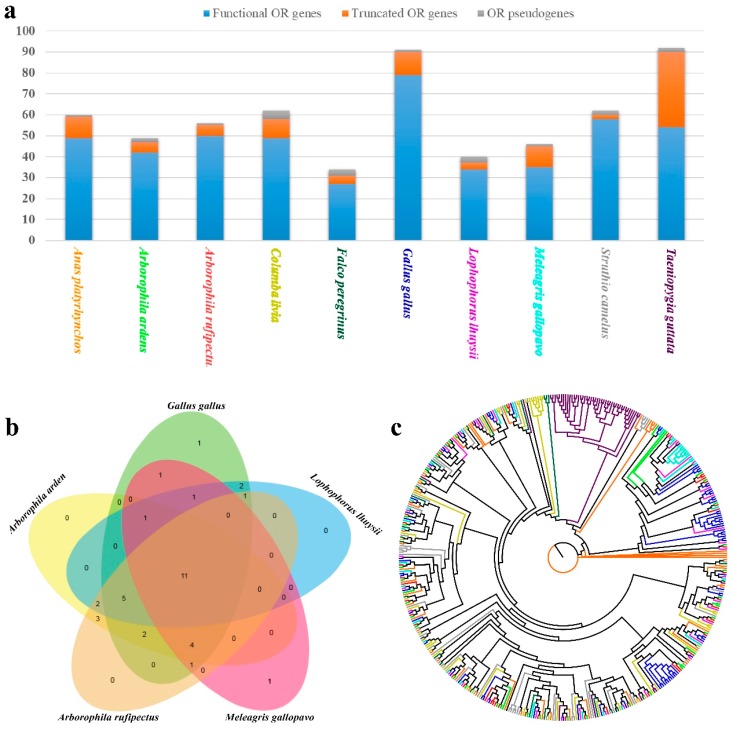
Identification of olfactory receptor (OR) genes and phylogenetic analysis. (**a**) Composition of OR genes in the ten avian species (**b**) Comparison of OR gene clusters among the five Phasianidae species. (**c**) The phylogenetic tree of OR functional genes from the ten bird genomes. Functional OR genes of each bird are represented by the same color as the species in (a).

**Figure 6:**
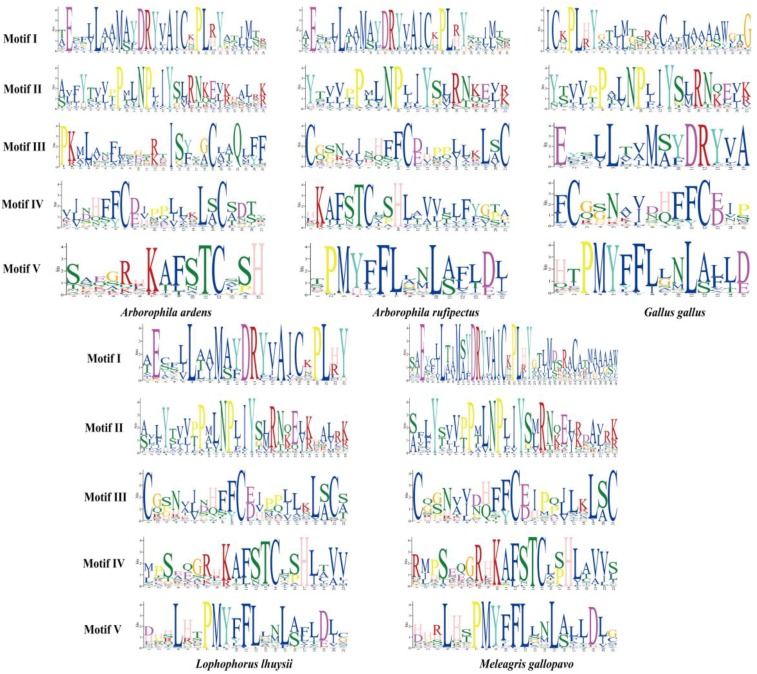
Logo representation of the five best conserved motifs identified for Phasianidae OR genes. The degree of conservation was represented by the height of the amino acid code.

The total number of OR genes varied from 34 to 92, with the average number of functional genes (47.7) considerably greater than truncated genes (9.5) or pseudogenes (2.0) (Figure 5a). The total number of OR genes was greatest in zebra finch, while the least in peregrine falcon. It was reported that OR genes with more than 60% identity in protein sequence are suggested to recognize odorants with related structures [33,34]. To evaluate the diversity in the OR gene repertoires of the ten studied birds, the identified avian OR functional genes were classified into families and subfamilies on the basis of phylogenetic analyses and their sequence similarity. The OR genes of the ten avian species were divided into 10 families and 80 subfamilies. The comparison of OR gene subfamilies of the five Phasianidae species is illustrated in Figure 5b. Phylogenetic comparison of OR repertoires suggested the obvious species-specific clustering pattern (Figure 5c), which was in line with previous studies [35,36]. Previous studies have indicated that some conserved amino acid motif features are found in mammalian OR genes [33,34,37]. To characterize the conserved motifs of OR protein sequences belonging to the five Phasianidae species, the five most conserved motifs were identified by the MEME program. As illustrated in Figure 6, the conserved motifs were strikingly similar among the Phasianidae species. The existence of the conserved motifs of OR genes has also been detected in other taxa (e.g., fish [38]).

### 3.4. Bird Phylogeny, Divergence and Evolution of Gene Families

A total of 14,668 gene families were identified for 10 bird species (Sichuan partridge, Hainan partridge, Chinese monal, turkey, chicken, mallard, peregrine falcon, zebra finch, ostrich) of which 5050 represented 1:1 orthologous gene families. We compared the orthologous gene clusters among five Phasianidae species (Sichuan partridge, Hainan partridge, Chinese monal, turkey, and chicken), which is shown in Figure 3b. The phylogenetic tree constructed based on the 1:1 orthologous genes suggested the basal phylogenetic position of the genus *Arborophila* within Phasianidae, and was most likely derived from a common ancestor approximately 49.1 million years ago (Mya) (Figure 3c).

### 3.5. Positive Selection in the Sichuan Partridge

**Figure 7:**
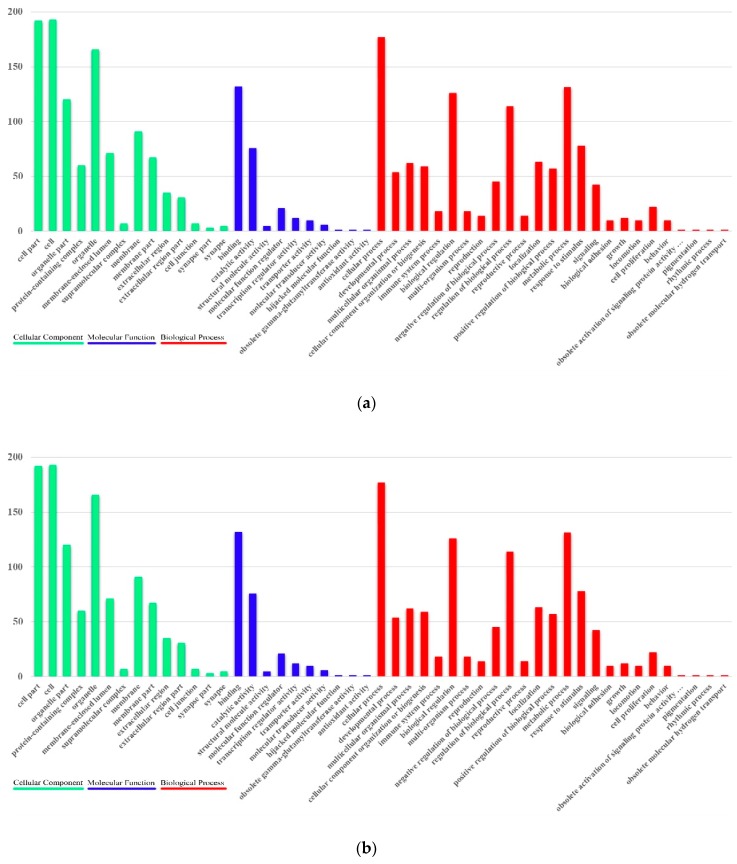
Functional distribution of positively selected genes (PSGs). (**a**) Functional distribution of PSGs according to the gene ontology (GO) database. The y-axis reveals the GO functional categories, while the number of genes in each category is plotted on the x-axis. (**b**) Functional distribution of PSGs according to the Kyoto Encyclopedia of Genes and Genomes (KEGG)pathway database. The y-axis illustrates the KEGG functional categories, while the number of genes in each category is plotted on the x-axis.

We found that 234 of the 5050 one-to-one orthologous genes were under positive selection in the Sichuan partridge. The Gene Ontology (GO) annotation classified the PSGs into three categories: molecular functions, cellular components, and biological processes (Figure 7a). Molecular functions included genes mainly involved in binding (132 genes; GO:0005488) and catalytic activity (76 genes; GO:0003824). Genes related to cellular components were primarily cell (193 genes; GO:0005623), cell part (192 genes; GO:0044464), and organelle (166 genes; GO:0043226). Biological process genes were mainly involved in cellular process (177 genes; GO:0009987), metabolic process (131 genes; GO:0008152), biological regulation (126 genes; GO:0065007), and regulation of biological process (114 genes; GO:0050789). The distribution of GO annotations in different functional categories showed a substantial diversity of PSGs. We identified biochemical pathways represented by the (PSGs). The KEGG annotation of the PSGs suggested that they were distributed in 40 pathways related to metabolism (24 genes), genetic information processing (19 genes), environmental information processing (19 genes), cellular processes (22 genes), organismal systems (17 genes), and human diseases (27 genes) (Figure 7b). We further performed GO enrichment with all the PSGs. GO enrichment identified significant overrepresentation of genes involved in environmental adaptation of Sichuan partridge (Table S6). Several pathways related to energy metabolism and behavior were found in Sichuan partridge, such as mitochondrion (GO:0005739), nitrogen compound metabolic process (GO:0006807), and adult locomotory behavior (GO:0008344).

### 3.6. Demography Reconstruction

**Figure 8:**
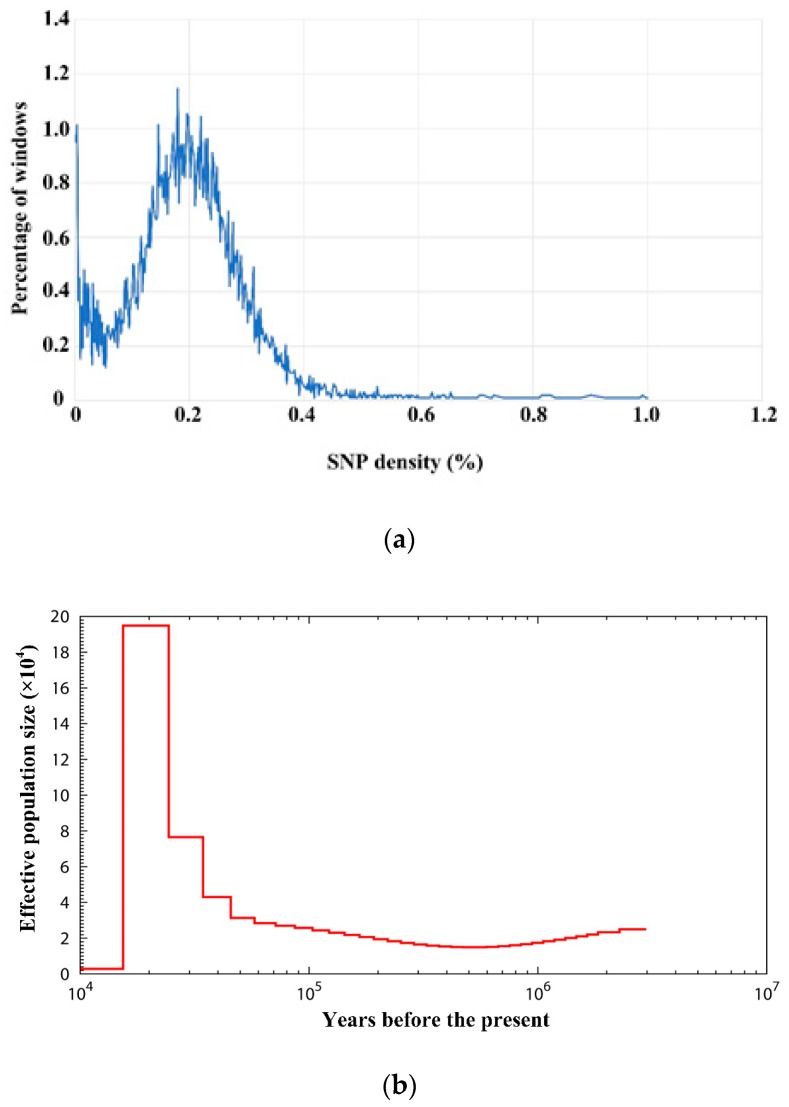
Single-nucleotide polymorphism (SNP) density distribution and demography reconstruction of Sichuan partridge. (**a**) Distribution of SNP density across Sichuan partridge genome. Heterozygous SNPs between the chromosomes were annotated, and heterozygosity density was observed in non-overlapping 50-kb windows. (**b**) Pairwise sequentially Markovian coalescent (PSMC) inference of Sichuan partridge population history on basis of autosomal data. The central bold lines represent inferred population sizes. The 100 thin curves surrounding each line are the PSMC estimates which were generated using 100 sequences randomly resampled from the original sequence. The mutation rate on autosomes used in time scaling, was estimated using chicken autosome data.

A total of 1,943,364 heterozygous SNPs in the Sichuan partridge genome were detected, and the genome-wide SNP density distribution is showed in Figure 8a. Pairwise sequentially Markovian coalescent modeling (PSMC) analysis was conducted based on local SNP densities to model the demographic history of Sichuan partridge from 10 million years ago to 10,000 years ago (Figure 8b). PSMC showed that the effective size of the Sichuan partridge population had experienced one sharp decrease from approximately 195,000 individuals to a minimum of 2500 individuals around 15,000 years ago. PSMC plots from a variety of birds [39] have revealed several taxa (e.g., the Rifleman) that underwent relatively drastic declines during the last glacial period. The Sichuan Partridge has a similar drastic decline. However, the Sichuan Partridge had a relatively small and stable population size for a long period of time prior to the increase to 195,000 individuals, which was then followed by a drastic decrease. This differs from the taxa that were observed to have a decrease in the prior study [39], which had fluctuating population sizes prior to the decline.

## 4. Discussion and Conclusions

The Sichuan partridge reference genome is an important resource for studying the genetic mechanisms and environmental adaptation facilitating more effective protection for this endangered species. The synteny analysis between the Sichuan partridge and chicken revealed the relatively conserved genome structures, which was consistent with previous reports of conserved overall synteny between ground tit and zebra finch [23], zebra finch and chicken [31], and also between turkey and chicken [40], suggesting a conserved genome structure among these avian species. However, further confirmation with more sequenced avian genomes is needed to be conducted for this inference.

Olfactory receptors (ORs) are expressed in sensory neurons within the olfactory epithelium and play a pivotal role in the sense of smell among vertebrates. To date, avian OR genes have been largely unexplored. In this study, an average number of 59 OR genes in the ten studied avian species were identified, and the total number of OR genes varied greatly from 34 to 92 across the bird species. The total number of OR functional genes in bird species was considerably fewer than in the mammals like pig (1113), cattle (881), the yak (981), and the forest musk deer (864) [37]. Among the five Phasianidae species, the total number of OR genes in the chicken was obviously greater than Sichuan partridge, Hainan partridge, turkey, and Chinese monal, which suggested that the olfaction capability of chicken was better than other Phasianidae birds. The enhanced olfaction capability of chicken can possibly be attributed to the domestication, which was in line with previous studies [37]. Pigs and cattle have been domesticated for thousands of years and selective breeding could have favored or enhanced olfaction capabilities. Previous study indicated that domesticated pig has highly developed noses for foraging in the soil [41], which revealed the important role of olfaction in a pig’s life. With a range of odiferous glands over their body and a strong sense of smell, cattle have been reported to be capable of recognizing companions, distinguishing gonads and receiving pheromones secreted by the skin [42]. The proportion of OR pseudogenes in the ten bird species was on average 3.90%, which was apparently less than in mammals like pig (14.5%), cattle (13.4%), yaks (34.8%), and forest musk deer (26.0%). The difference in the number of OR genes and the percentage of OR pseudogene may be attributed to the difference of survival strategies, the quality of the genomes and/or different bioinformatics search strategies used. Furthermore, the genome quality was reported to possibly contribute to the difference in the proportion of OR pseudogenes [36]. Therefore, a more accurate analysis of OR gene repertoires in these ten species, and whether the olfaction capability of chicken was more highly developed than other birds needs verification.

The phylogenetic analysis revealed the monophyly of the Phasianidae and the genus *Arborophila* was given a basal phylogenetic position, branching apparently earlier than other genera within Phasianidae. This result was consistent with the results of previous phylogenetic analyses that have also placed *Arborophila* basal to the balance of phasianines that we included in our analysis [43,44,45,46]. The genus *Arborophila* diverged from the other lineages in the Phasianidae around 49.1 Mya, which was much earlier than other genera [43].

The altitude of Laojunshan National Nature Reserve ranges from 1100 to 2008 m and has an annual average temperature of 12.5 °C [4]. Previous studies reported that Sichuan partridge eggs experienced an average of 4.2 h per day below 26 °C (the ‘Physiological Zero Temperature’) at which the embryo cannot develop [47]. Although the temperature is fairly more than that reported for most birds and likely to cause death of the embryo, the hatching success of Sichuan partridge was 88.4%, which is approaching the average hatchability rate for birds generally (89.1%) [47]. The positively selected genes, found in this study, related to energy metabolism possibly illustrated the molecular genetic mechanisms of adaptation to cold montane environments and embryonic tolerance of hypothermia of Sichuan partridge. Compared to birds tending to nest in relatively quieter areas, Sichuan partridge was reported to nest in more disturbed areas, but hatching success was not reduced [47]. Nesting located close to forest trails is likely to gain some advantages because disturbance along trails could reduce predation to some extent. At the very least, reference [47] has illustrated the resilience of the Sichuan partridge to disturbance along forest trails and revealed that increased visitor numbers to Laojunshan National Nature Reserve did not necessarily result in reduced breeding success of Sichuan partridge, and that the reserve is there to protect. Energy and behavior related pathways analyzed in this study will provide pivotal information for understanding the molecular genetic mechanism of the special reproduction of Sichuan partridge.

In summary, this is the first report describing the complete Sichuan partridge genome. We illustrated the conserved genome structure between the Sichuan partridge and chicken and the basal phylogenetic position of the genus *Arborophila* within Phasianidae. We used the PSMC to infer the demographic history of the Sichuan partridge on the basis of the distribution of heterozygote sites across the genome. Positively selected genes related to energy metabolism and behavior possibly hint the molecular genetic mechanisms of the environmental adaptation of the Sichuan partridge. Further research is needed to further explore the significance of the findings presented here. The Sichuan partridge reference genome will play an instrumental role in the future examination of adaptive evolution to the environment and aiding the long-term conservation of this endangered species and their genetic diversity. Our de novo assembled genome presented here will provide a resource for the future examination of evolution and adaptation of Sichuan Partridge, and the genome will eventually be useful in aiding the long-term conservation of Sichuan Partridge.

## Figures and Tables

**Table 1:** Genome sequencing information of the Sichuan partridge.

Insert Size (bp)	Read Length (bp)	Raw Data			Clean Data		
		Read Number	Total Bases (Gb)	Sequencing Depth (x)	Read Number	Total Bases (Gb)	Sequencing Depth (x)
250	150	485,619,976	72.84	66.80	476,794,840	71.52	65.62
500	150	267,992,818	40.20	36.88	263,165,590	39.47	36.22
800	150	423,275,628	63.49	58.25	414,846,834	62.23	57.09
2000	150	207,573,892	31.14	28.57	167,760,376	25.16	23.09
5000	150	433,978,936	65.10	59.73	408,121,682	61.22	56.17
10,000	150	265,968,600	39.90	36.60	231,043,688	34.66	31.80
15,000	150	96,333,412	14.45	13.26	96,333,412	14.45	13.26
20,000	150	138,791,494	20.82	19.10	98,033,406	14.71	13.49

Estimated genome size of the Sichuan partridge is 1.09 Gb.

**Table 2:** Statistics of repetitive elements in five Phasianidae species.

Type	*A. rufipectus*		*A. ardens*		*G. gallus*		*M. gallopavo*		*L. lhuysii*	
	Length (bp)	Percentage (%)	Length (bp)	Percentage (%)	Length (bp)	Percentage (%)	Length (bp)	Percentage (%)	Length (bp)	Percentage (%)
SINEs	531,088	0.05	532,392	0.05	611,338	0.05	565,262	0.05	557,053	0.06
LINEs	69,173,064	6.35	67,925,543	6.50	81,486,693	6.62	82,214,258	7.29	70,584,167	6.97
LTR elements	19,342,763	1.77	13,540,223	1.30	26,993,586	2.19	23,893,383	2.12	14,107,359	1.39
DNA elements	11,459,489	1.05	11,554,622	1.11	13,381,436	1.09	11,677,720	1.03	11,403,896	1.13
Unclassified	716,293	0.07	767,625	0.07	1,772,312	0.14	1,182,696	0.10	632,586	0.06
Total	101,222,697	9.29	94,320,405	9.02	124,245,365	10.10	119,533,319	10.59	97,285,061	9.61

**Table 3:** The summary of perfect microsatellite types in five Phasianidae species.

Types	*A. rufipectus*			*A. ardens*			*G. gallus*			*M. gallopavo*			*L. lhuysii*		
	Counts	Length (bp)	Percentage (%)	Counts	Length (bp)	Percentage (%)	Counts	Length (bp)	Percentage (%)	Counts	Length (bp)	Percentage (%)	Counts	Length (bp)	Percentage (%)
Mono-	263,050	4,738,050	71.58	317,580	5,538,023	74.56	216,419	3,462,413	52.44	140,879	1,959,747	13.91	209,830	3,535,260	71.75
Di-	23,602	427,292	6.42	24,677	457,440	5.79	31,485	590,712	7.63	26,237	584,466	22.28	20,669	376,944	7.07
Tri-	22,277	402,633	6.06	23,886	445,965	5.61	27,148	487,461	6.58	20,303	446,127	21.97	18,649	335,742	6.38
Tetra-	42,231	896,888	11.49	42,527	968,896	9.98	47,327	1,114,116	11.47	37,434	814,944	21.77	29,203	611,568	9.99
Penta-	14,233	460,685	3.87	15,035	572,380	3.53	55,616	2,187,015	13.48	13,247	418,520	31.59	11,498	500,615	3.93
Hexa-	2120	84,402	0.58	2251	92,466	0.53	34,679	1,610,112	8.4	3390	113,010	33.34	2581	105,420	0.88

## Supplementary Materials

The following are available online at https://www.mdpi.com/2073-4425/10/9/677/s1, Table S1: Statistics of the genome completeness of the Sichuan partridge based on 248 CEGs, Table S2: Statistics of the genome completeness of Sichuan partridge based on BUSCO benchmark, Table S3: Functional annotation statistics of the Sichuan partridge genome, Table S4: The number and frequency of the SSRs in the Sichuan Partridge genome, Table S5: The most abundant motif categories found in the Sichuan partridge genome, Table S6: GO enrichment of the PSGs in the Sichuan partridge, Figure S1: The alignment of Sichuan partridge scaffolds to the chicken reference genome.
