# Supplementary material for: The Draft Genome of the Endangered Sichuan Partridge (Arborophila rufipectus) with Evolutionary Implications
Source: Genes (Basel). 2019 Sep 5;10(9):677. doi: 10.3390/genes10090677 (PMC6770966; doi:10.3390/genes10090677)
Supplement: Supplementary file 1 [file genes-10-00677-s001.pdf]

Table S1. Statistics of the genome completeness of the Sichuan partridge based on 248 CEGs.

	Prots	Completeness
Completeness	192	77.42
Group1	48	72.73
Group2	47	83.93
Group3	45	73.77
Group4	52	80.00
Partial	213	85.89
Group1	54	81.82
Group2	51	91.07
Group3	51	83.61
Group4	57	87.69

Table S2. Statistics of the genome completeness of Sichuan partridge based on BUSCO benchmark.

BUSCO benchmark	Number	Percentage
Complete Single-Copy BUSCOs	262	86.5
Complete Duplicated BUSCOs	9	3.0
Fragmented BUSCOs	13	4.3
Missing BUSCOs	19	6.2
Total BUSCO groups searched	303	100

Table S3. Functional annotation statistics of the Sichuan partridge genome.

		Number	Percentage (%)
Total		17519	100
	SwissProt	15080	86.08
	TrEMBL	15704	89.64
	KEGG	9201	52.52
Annotated	InterPro	15264	87.13
	GO	14585	83.25
	Nr	15680	89.50
	all	15891	90.71
Unannotated		1628	9.29

Table S4. The number and frequency of the SSRs in the Sichuan Partridge genome.

	P-SSRs	CD-SSRs	IP-SSRs
No. of SSRs	367,513	22,009	1,059,066
Relative abundance (loci/Mb)	358.35	21.46	1,032.65
Relative density (bp/Mb)	6,835.09	1,087.95	32,487.82

P perfect, CD compound, IP imperfect

Table S5. The most abundant motif categories found in the Sichuan partridge genome.

Motif	Counts	Length(bp)	Percent (%)	Average Length(bp)	Relative Abundance (loci/Mb)	Relative Density (bp/Mb)
A	246777	4476861	67.15	18.14	240.62	4365.19
C	16273	261189	4.43	16.05	15.87	254.67
AC	8445	158296	2.3	18.74	8.23	154.35
AG	4248	77494	1.16	18.24	4.14	75.56
AT	10885	191134	2.96	17.56	10.61	186.37
CG	24	368	0.01	15.33	0.02	0.36
AAC	7629	140757	2.08	18.45	7.44	137.25
AAG	1292	24324	0.35	18.83	1.26	23.72
AAT	7301	132795	1.99	18.19	7.12	129.48
ACC	753	12618	0.2	16.76	0.73	12.3
ACG	742	12534	0.2	16.89	0.72	12.22
AAAC	15368	285576	4.18	18.58	14.98	278.45
AAAG	4150	121652	1.13	29.31	4.05	118.62
AAAT	14965	295924	4.07	19.77	14.59	288.54
AACC	613	10672	0.17	17.41	0.6	10.41
AACG	49	848	0.01	17.31	0.05	0.83
AAAAC	4395	106815	1.2	24.3	4.29	104.15
AAAAG	1450	73920	0.39	50.98	1.41	72.08
AAAAT	1834	49095	0.5	26.77	1.79	47.87
AAACC	202	4965	0.05	24.58	0.2	4.84
AAACG	57	1300	0.02	22.81	0.06	1.27
AAAAAC	405	11772	0.11	29.07	0.39	11.48
AAAAAG	318	13908	0.09	43.74	0.31	13.56

AAAAAT	165	4722	0.04	28.62	0.16	4.6
AAAACC	13	324	0	24.92	0.01	0.32
AAAAGG	4	102	0	25.5	0	0.1

Table S6. GO enrichment of the PSGs in the Sichuan partridge.

GO Term	GO ID	No. of genes	Corrected P-Value
organelle	GO:0043226	55	4.95E-05
intracellular organelle	GO:0043229	51	4.95E-05
membrane-bounded organelle	GO:0043227	50	4.95E-05
cell part	GO:0044464	62	4.95E-05
cell	GO:0005623	62	4.95E-05
intracellular membrane-bounded organelle	GO:0043231	46	5.43E-05
cellular process	GO:0009987	58	0.000105443
intracellular part	GO:0044424	54	0.000128653
intracellular organelle part	GO:0044446	35	0.00024631
primary metabolic process	GO:0044238	43	0.00026876
intracellular	GO:0005622	54	0.000308954
organelle part	GO:0044422	35	0.000308954
cytoplasmic part	GO:0044444	32	0.000691832
metabolic process	GO:0008152	45	0.000691832
organic substance metabolic process	GO:0071704	43	0.000691832
catalytic activity	GO:0003824	31	0.000874291
single-organism process	GO:0044699	48	0.001015162
cellular metabolic process	GO:0044237	41	0.001060523
chromosomal region	GO:0098687	7	0.001060523
mitochondrion	GO:0005739	14	0.001060523
macromolecule methylation	GO:0043414	6	0.00214307
cytoplasm	GO:0005737	38	0.006069098
chromosome, centromeric region	GO:0000775	5	0.006906682
adult locomotory behavior	GO:0008344	4	0.007289874

methylation	GO:0032259	6	0.007289874
single-organism cellular process	GO:0044763	40	0.008678494
macromolecular complex	GO:0032991	24	0.010021301
macromolecule metabolic process	GO:0043170	34	0.012500235
transferase activity, transferring one-carbon groups	GO:0016741	5	0.013710017
cellular macromolecule metabolic process	GO:0044260	32	0.013806377
biological regulation	GO:0065007	38	0.014949188
macromolecule modification	GO:0043412	19	0.014949188
adult behavior	GO:0030534	4	0.015199625
centrosome duplication	GO:0051298	3	0.017531868
microtubule polymerization	GO:0046785	3	0.017531868
gene silencing	GO:0016458	4	0.019672315
chromosomal part	GO:0044427	8	0.021556
protein complex	GO:0043234	20	0.025252551
peptidyl-lysine modification	GO:0018205	5	0.025252551
behavior	GO:0007610	6	0.027391061
organelle organization	GO:0006996	17	0.027391061
chromosome	GO:0005694	8	0.029718259
alpha-amino acid metabolic process	GO:1901605	4	0.030056357
centrosome cycle	GO:0007098	3	0.03202339
single-organism behavior	GO:0044708	5	0.032226173
nitrogen compound metabolic process	GO:0006807	25	0.037771357
positive regulation of microtubule polymerization or depolymerization	GO:0031112	2	0.037771357
neurotransmitter metabolic process	GO:0042133	2	0.037771357
microtubule polymerization or depolymerization	GO:0031109	3	0.037771357
chromatin silencing	GO:0006342	3	0.037771357

nucleic acid binding	GO:0003676	18	0.037771357
protein metabolic process	GO:0019538	22	0.037771357
binding	GO:0005488	40	0.037771357
microtubule-based process	GO:0007017	6	0.037771357
locomotory behavior	GO:0007626	4	0.037771357
negative regulation of gene expression, epigenetic	GO:0045814	3	0.037771357
intracellular organelle lumen	GO:0070013	16	0.037771357
organelle lumen	GO:0043233	16	0.037771357
membrane-enclosed lumen	GO:0031974	16	0.037771357
nucleus	GO:0005634	25	0.037789302
macromolecular complex subunit organization	GO:0043933	12	0.037808445
molecular function regulator	GO:0098772	8	0.039006363
microtubule cytoskeleton organization	GO:0000226	5	0.039969645
organic substance catabolic process	GO:1901575	10	0.039969645
covalent chromatin modification	GO:0016569	5	0.040282152
centrosome organization	GO:0051297	3	0.040282152
methyltransferase activity	GO:0008168	4	0.040282152
catabolic process	GO:0009056	10	0.040282152
organonitrogen compound catabolic process	GO:1901565	4	0.041467433
cellular protein metabolic process	GO:0044267	20	0.043575526
microtubule organizing center organization	GO:0031023	3	0.045048675
single-organism metabolic process	GO:0044710	17	0.048669588

Figure S1. The alignment of Sichuan partridge scaffolds to the chicken reference genome.

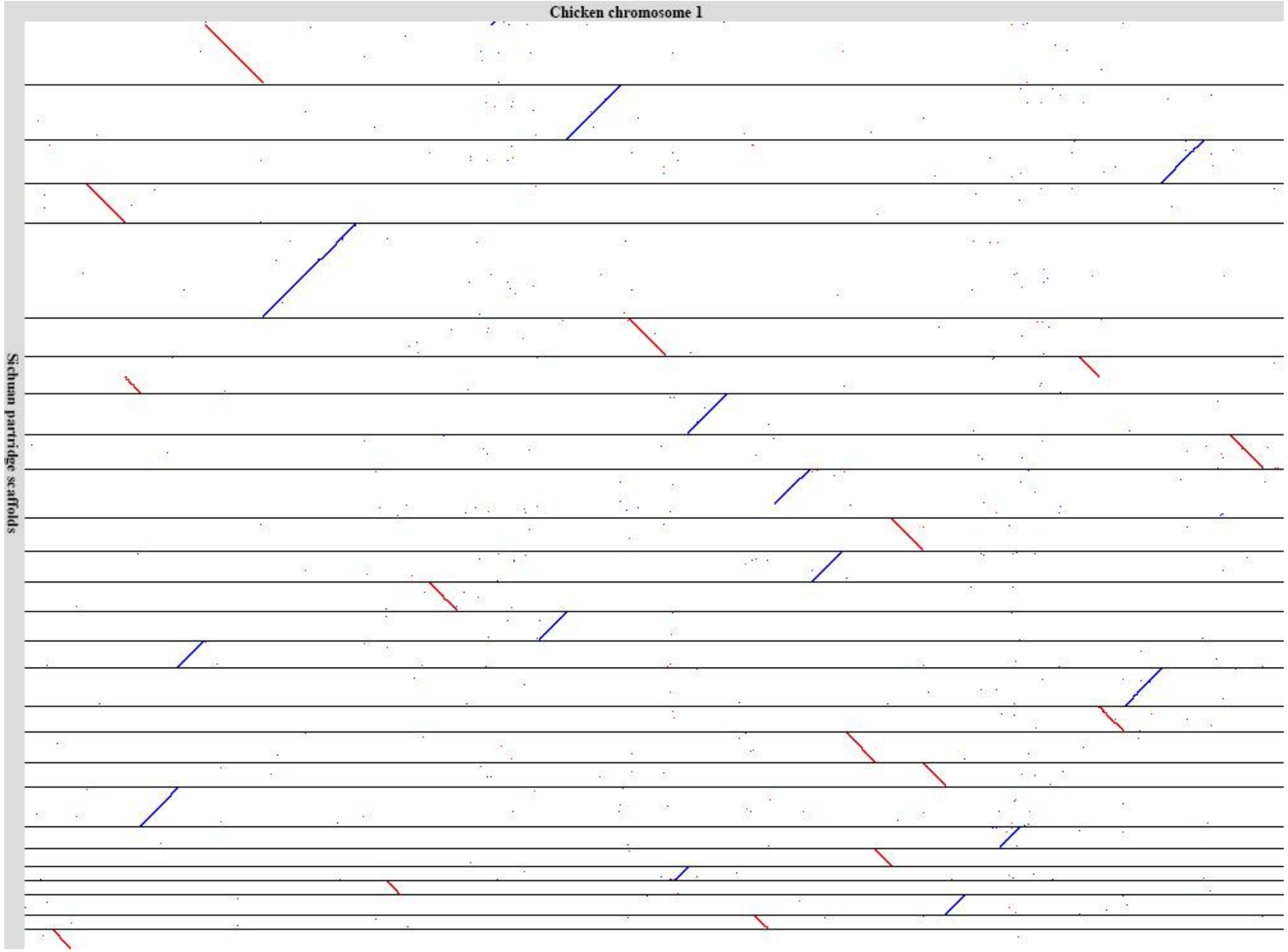

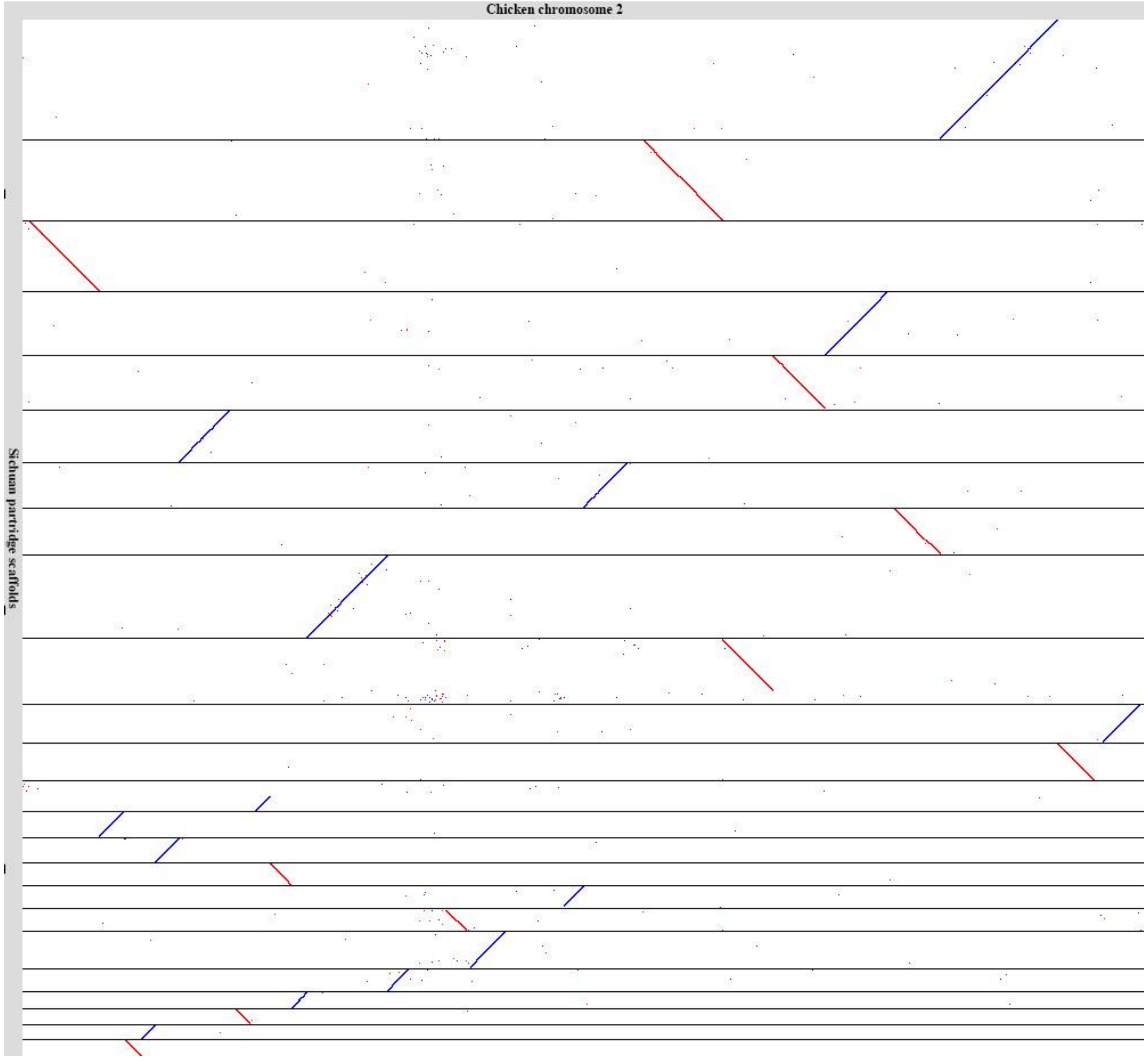

Chicken chromosome 2
Sichuan partridge scaffolds

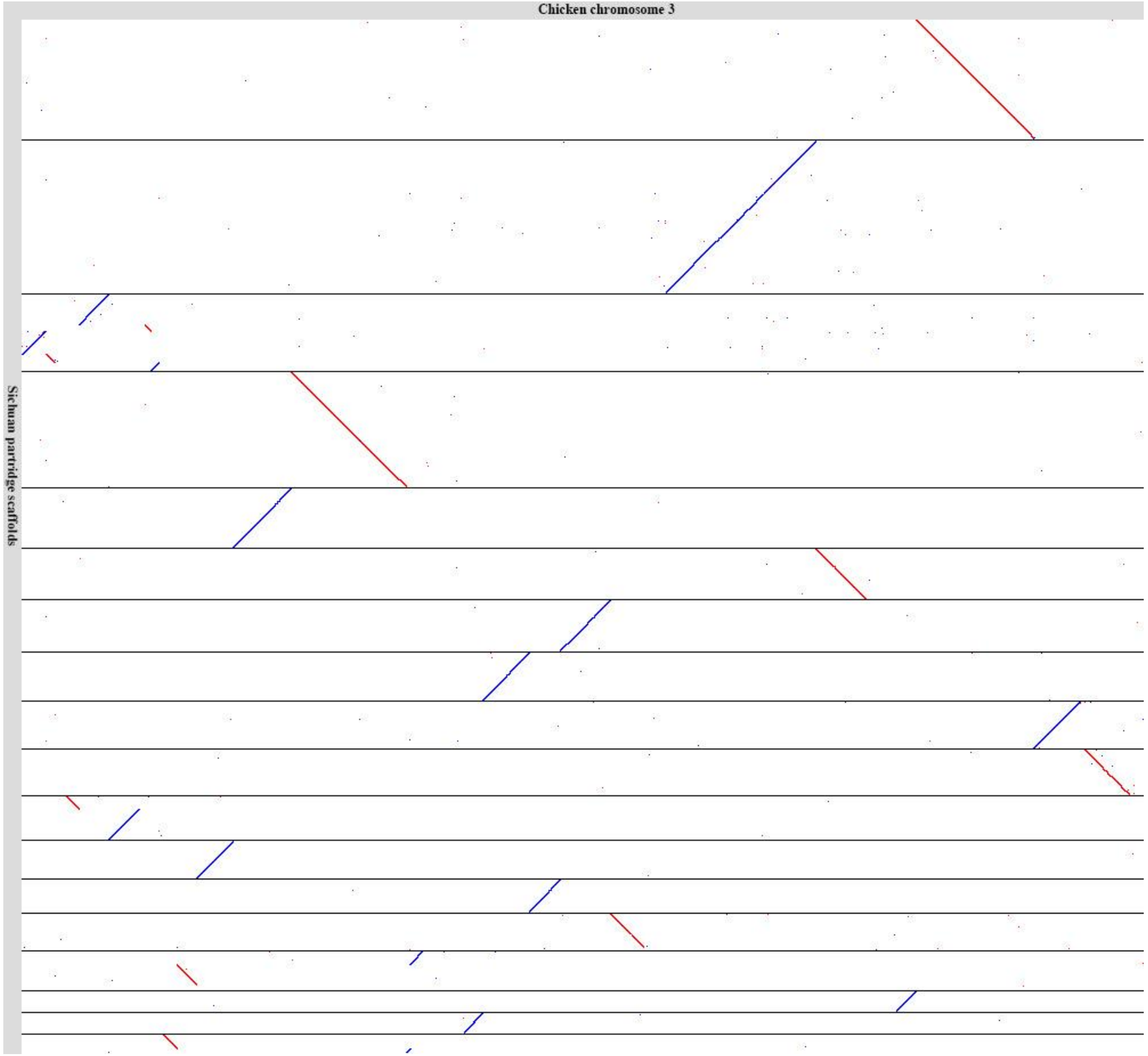
Chicken chromosome 3
Sichuan partridge scaffolds

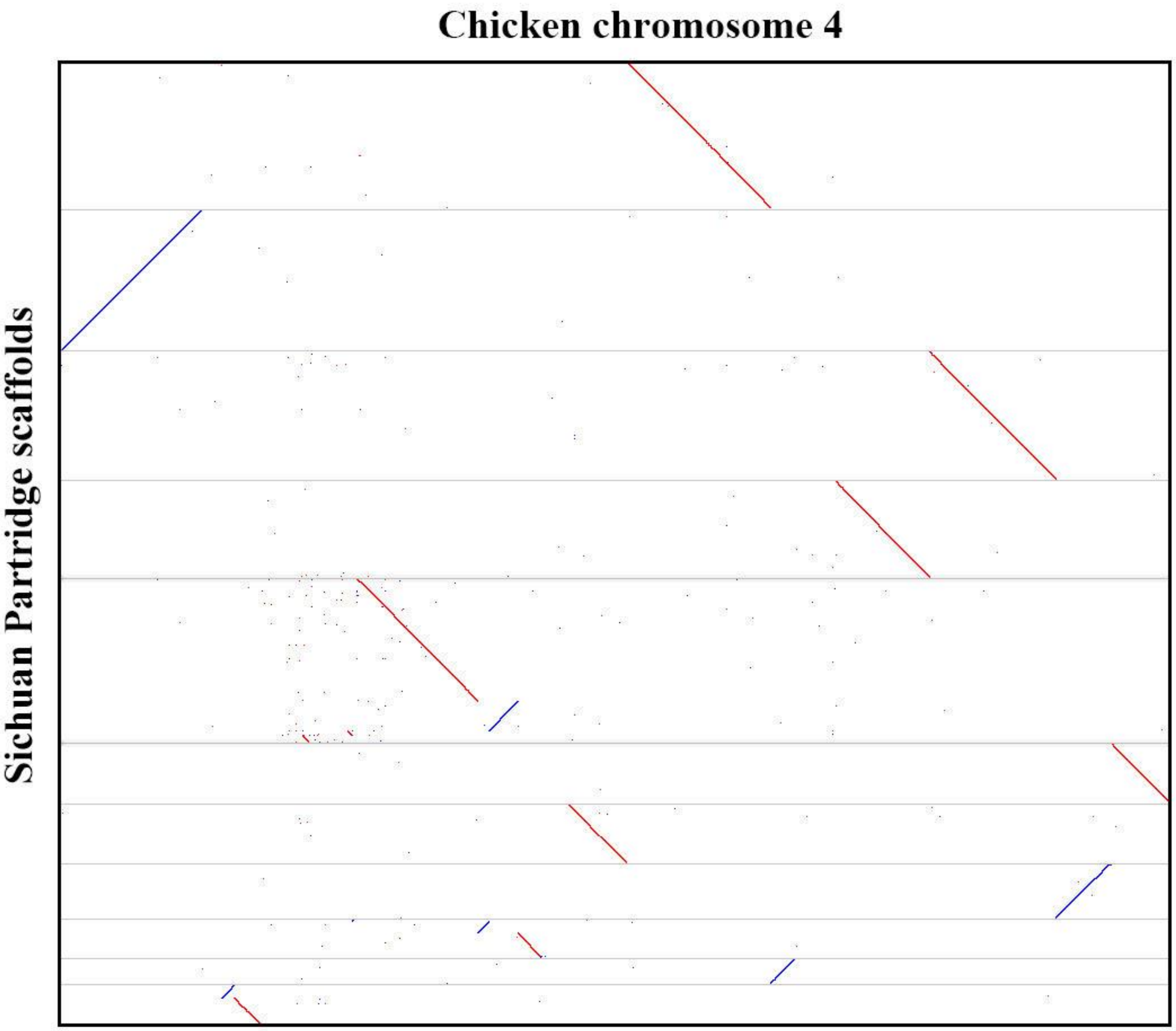

Chicken chromosome 4
Sichuan Partridge scaffolds

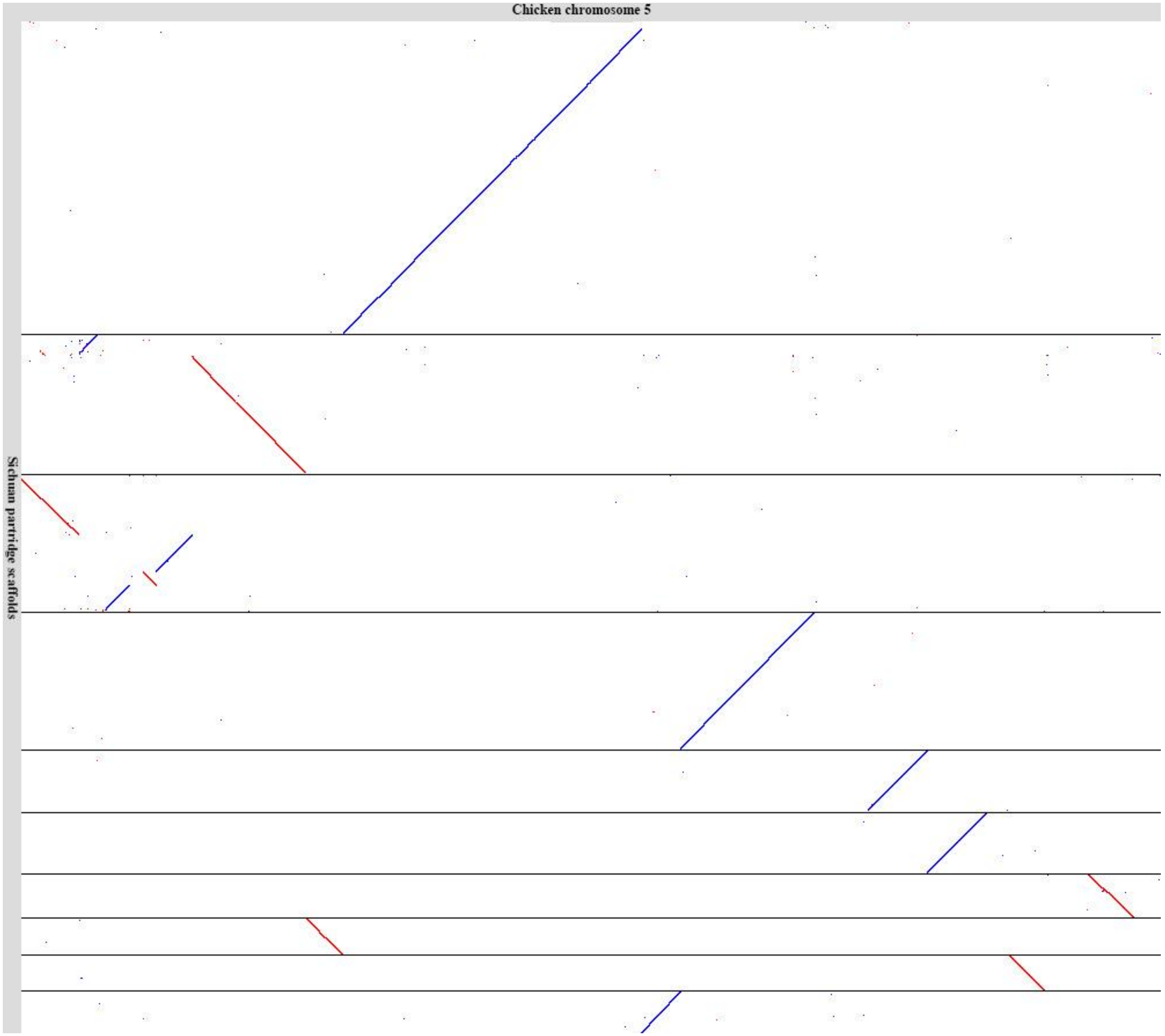

Chicken chromosome 5
Sichuan partridge scaffolds

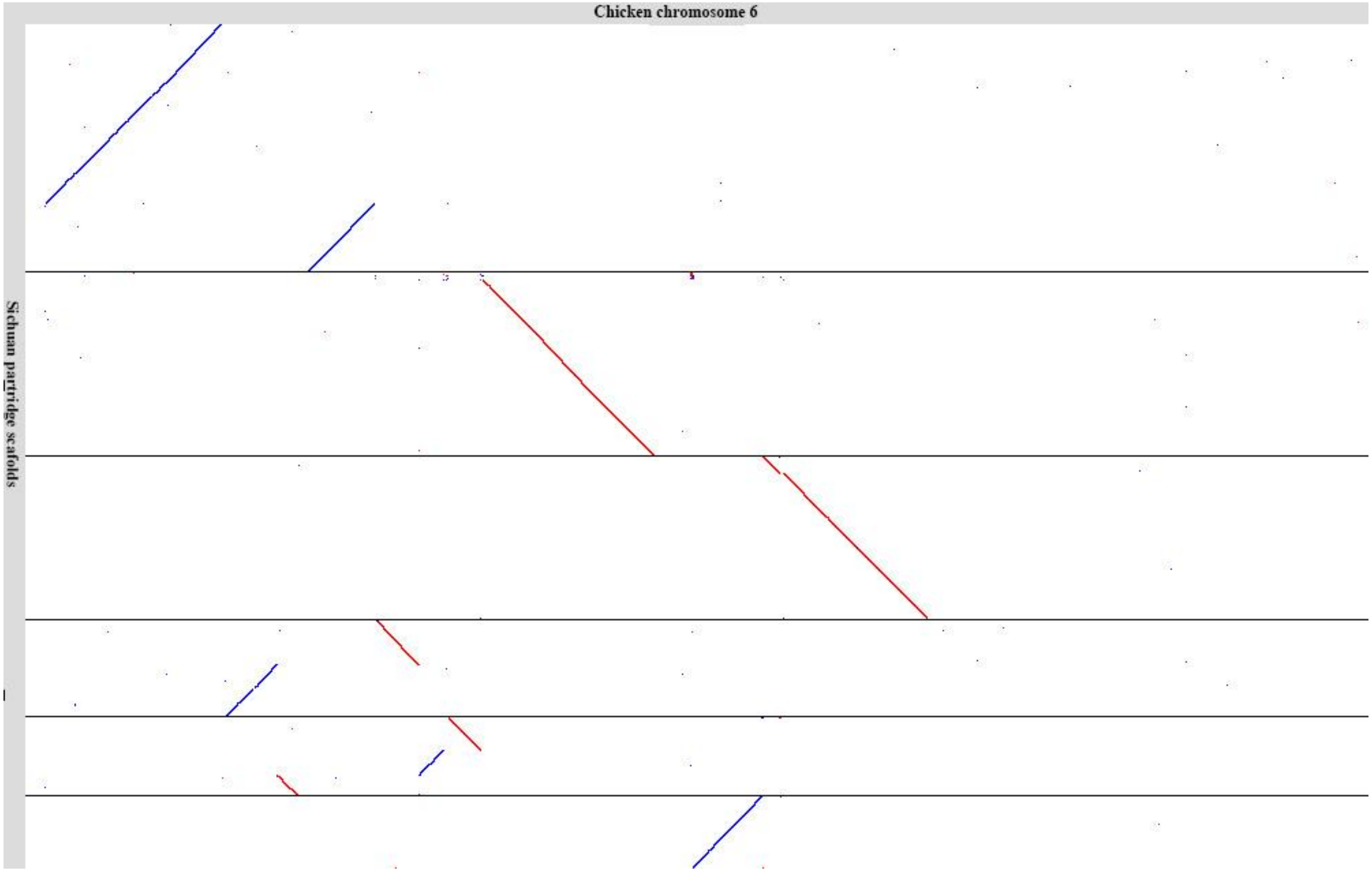
Chicken chromosome 6
Sichuan partridge scafolds

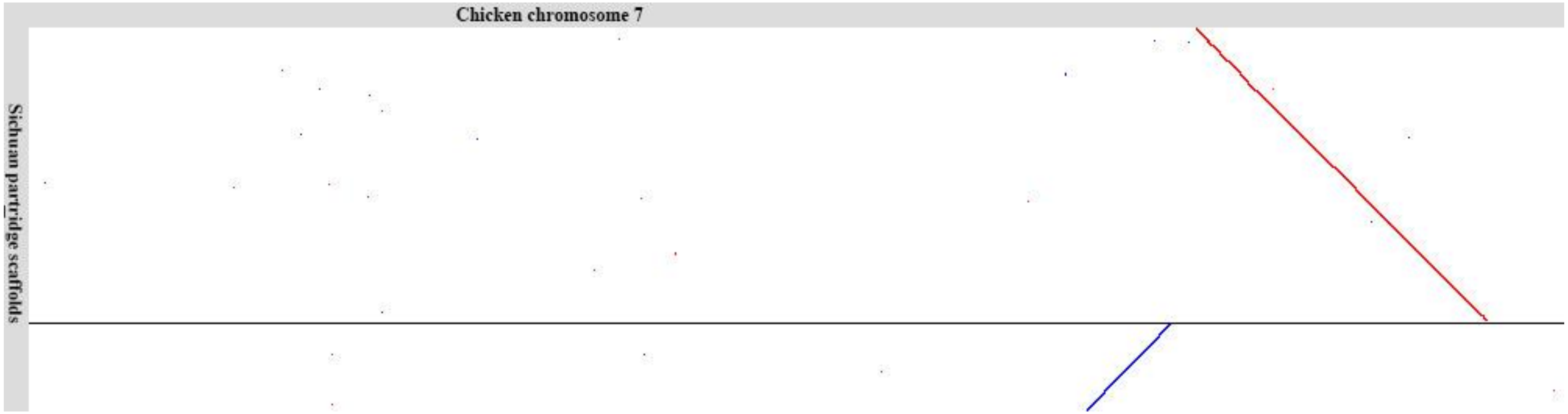
Chicken chromosome 7
Sichuan partridge scaffolds

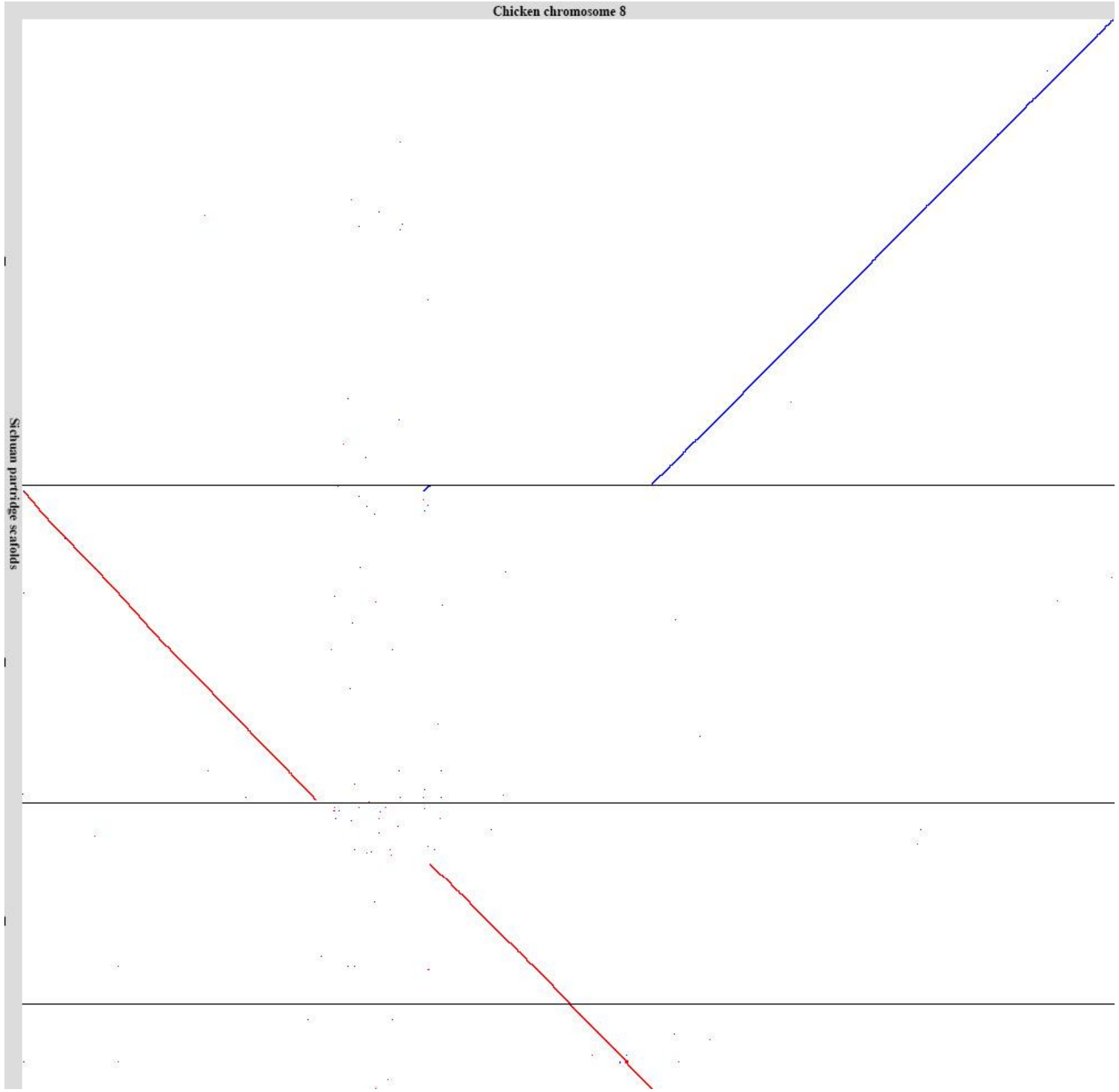
Chicken chromosome 8
Sichuan partridge scafolds

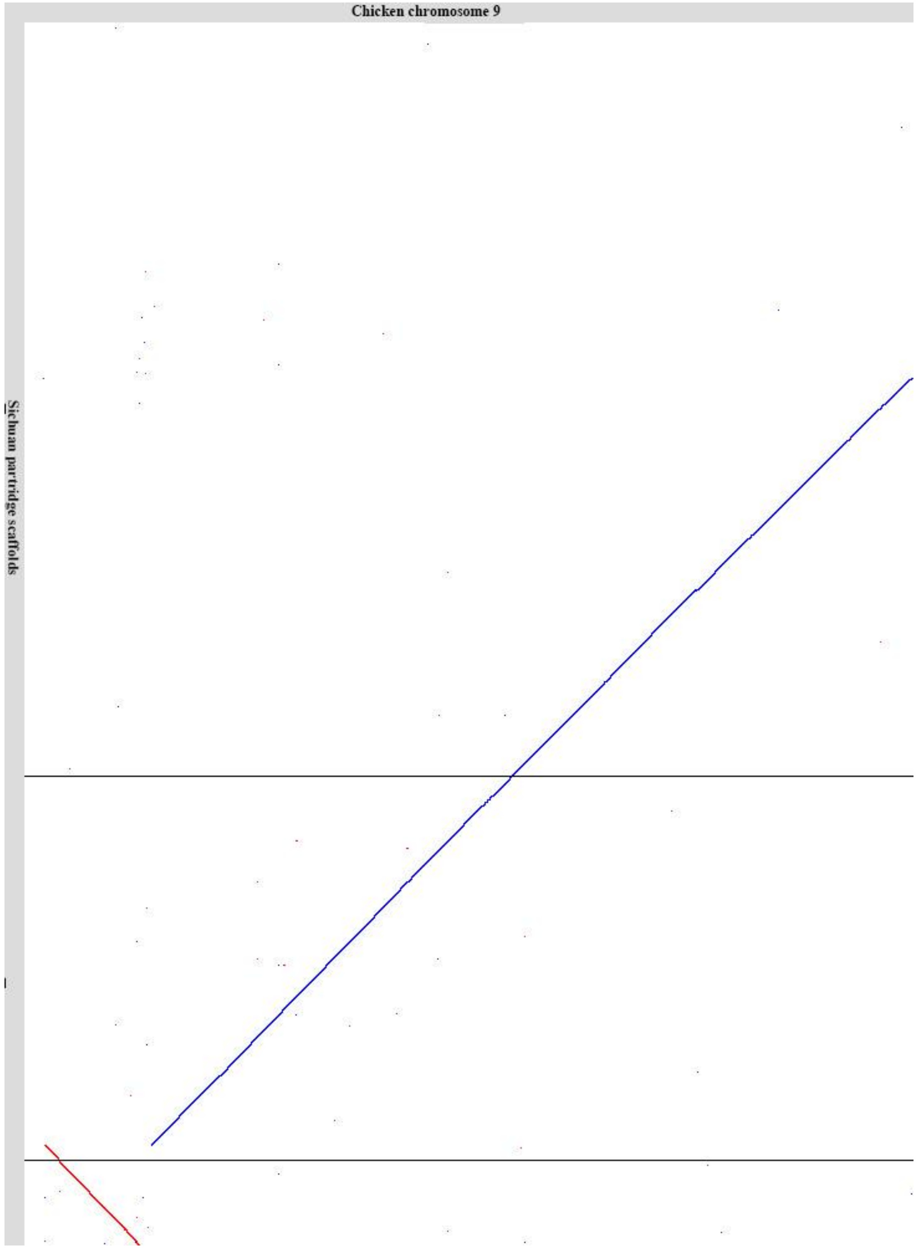
Chicken chromosome 9
Sichuan partridge scaffolds

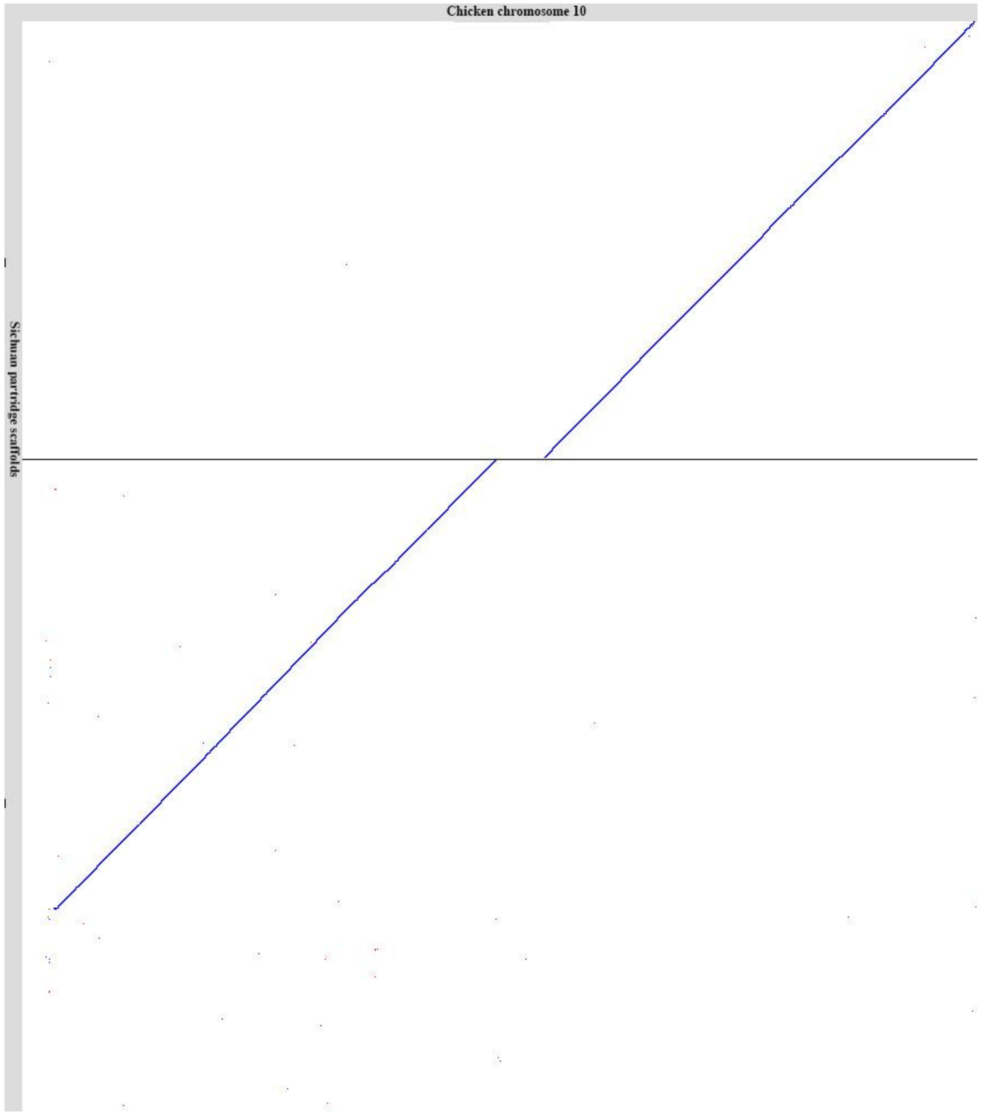
Chicken chromosome 10
Sichuan partridge scaffolds

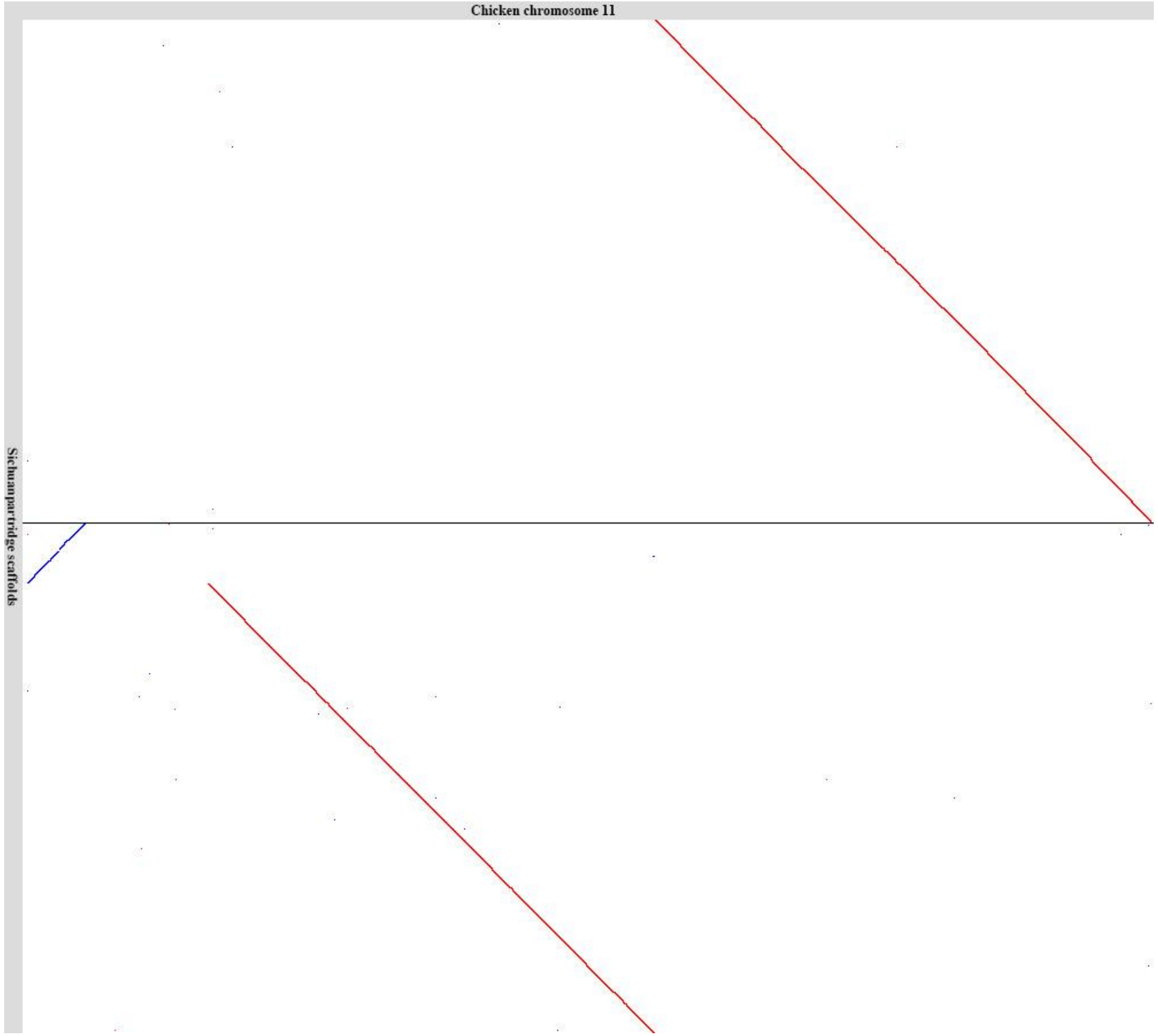
Chicken chromosome 11
Sichuanpartridge scaffolds

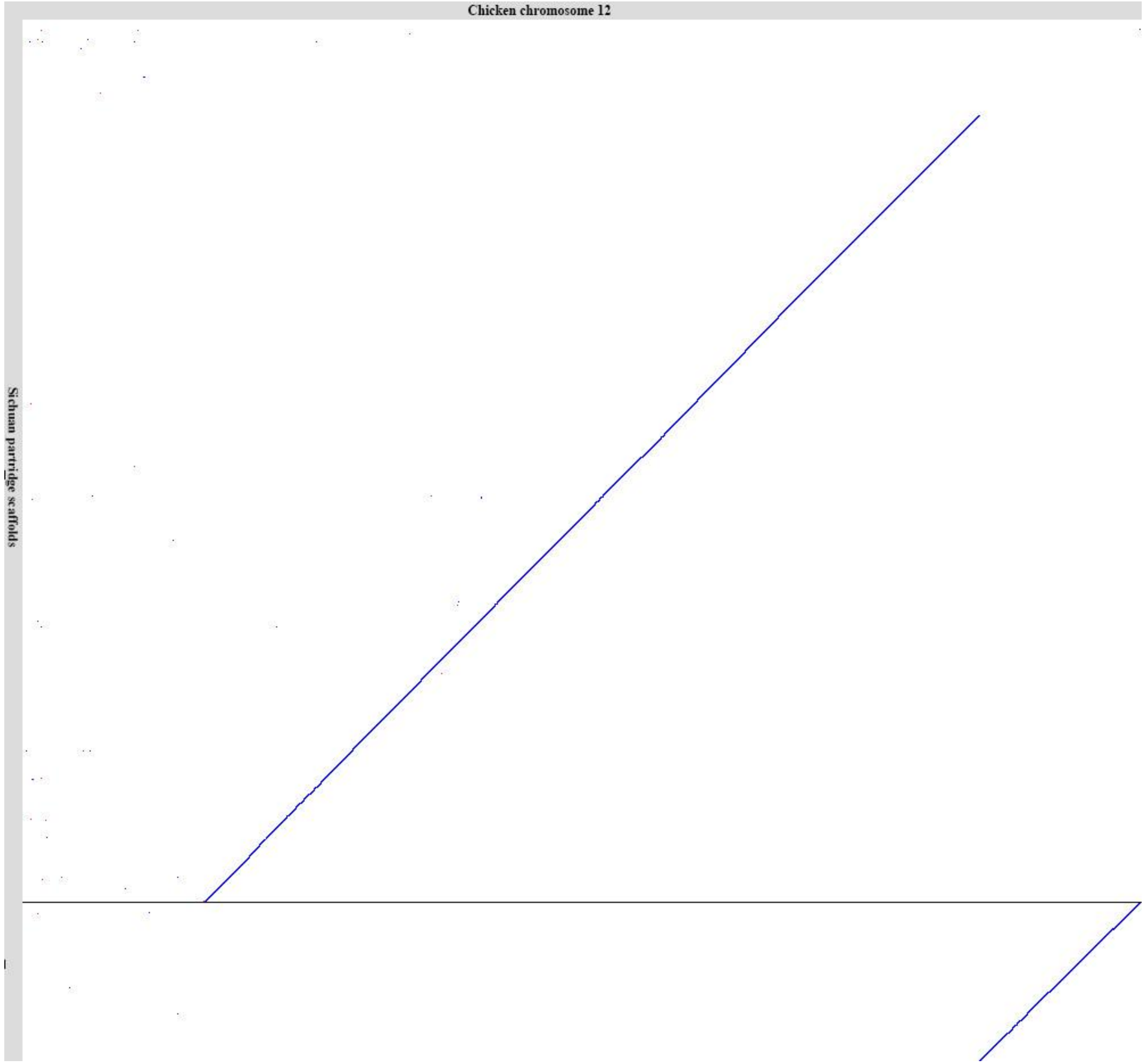

Chicken chromosome 12
Sichuan partridge scaffolds

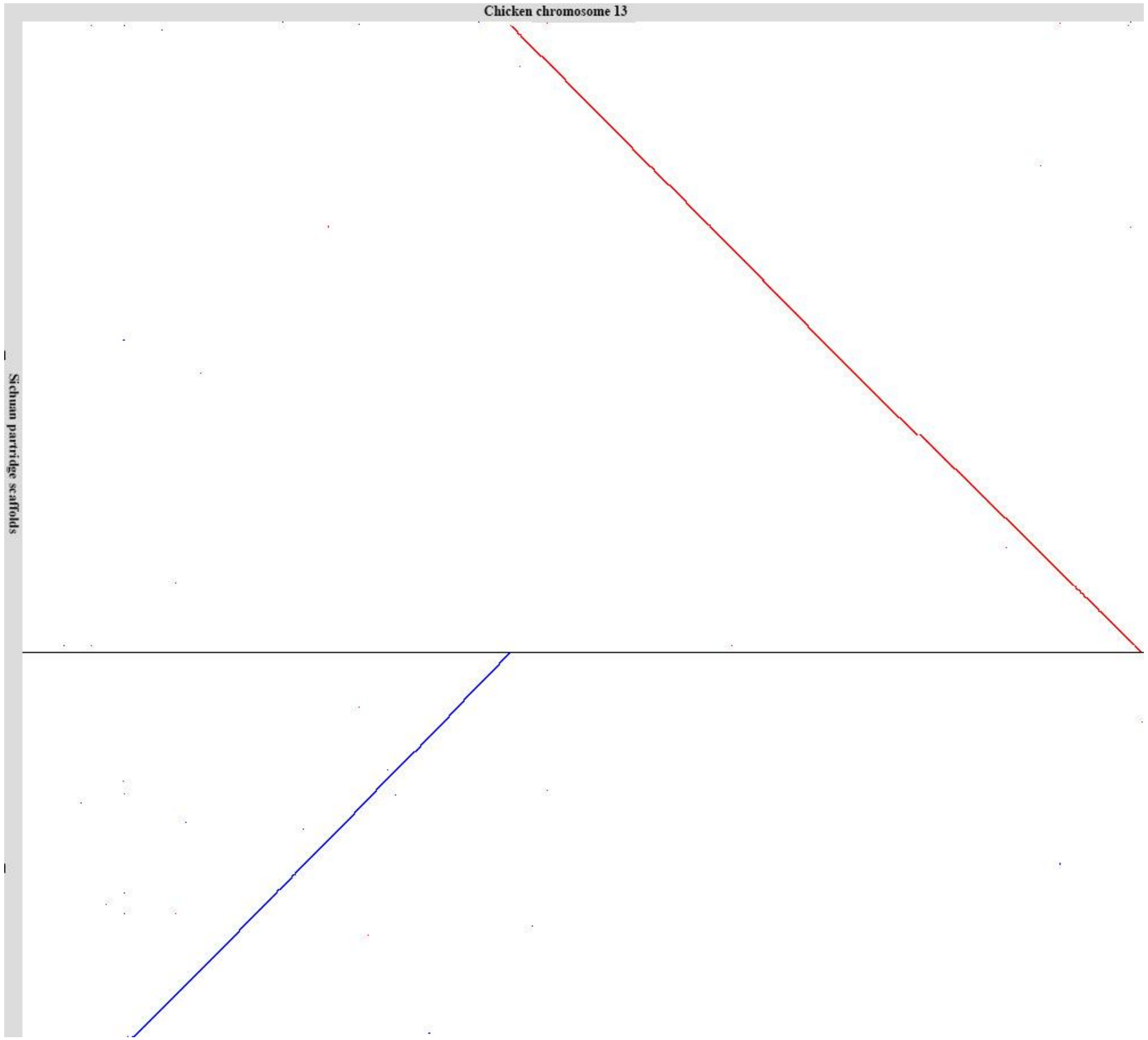
Chicken chromosome 13
Sichuan partridge scaffolds

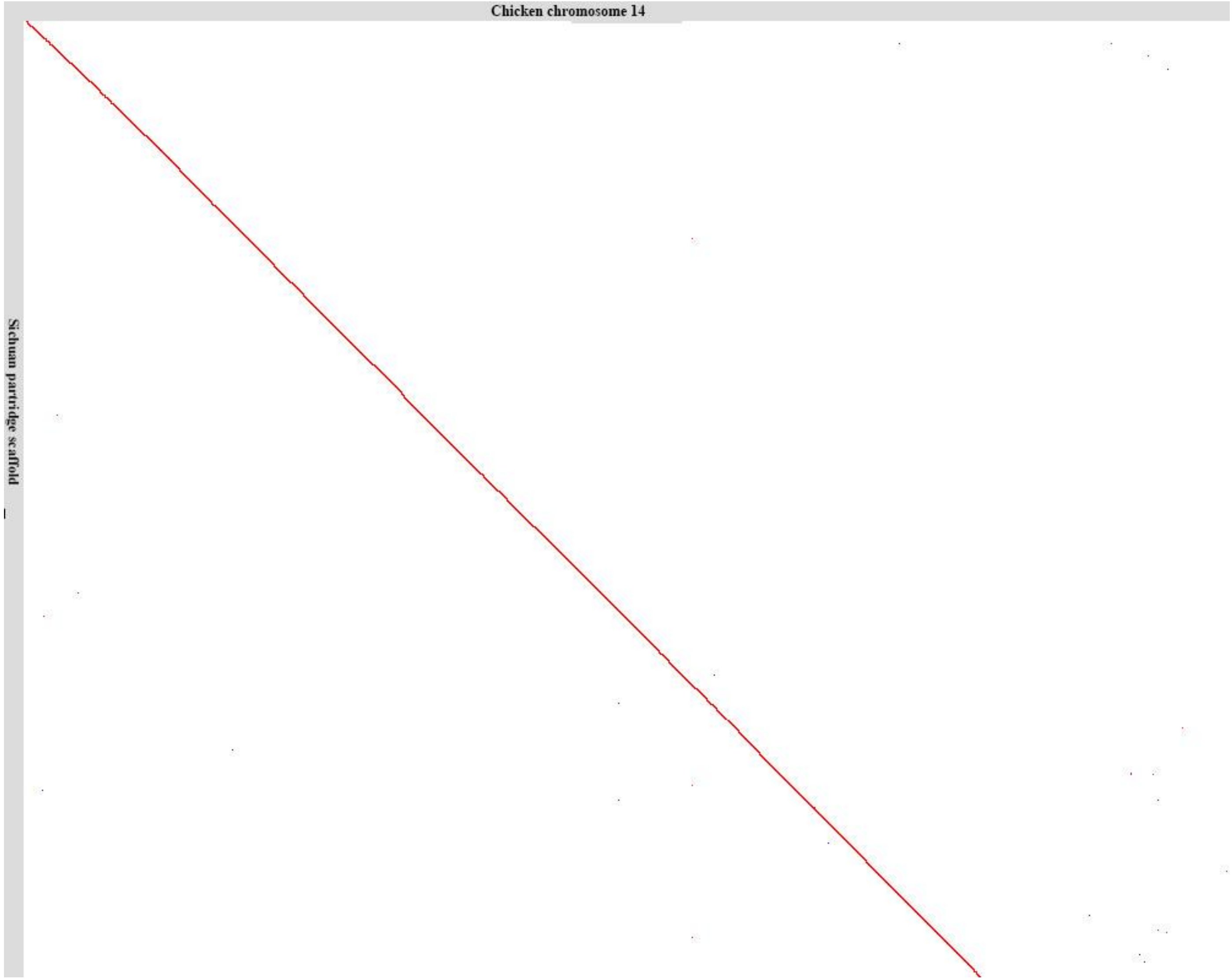
Chicken chromosome 14
Sichuan partridge scaffold

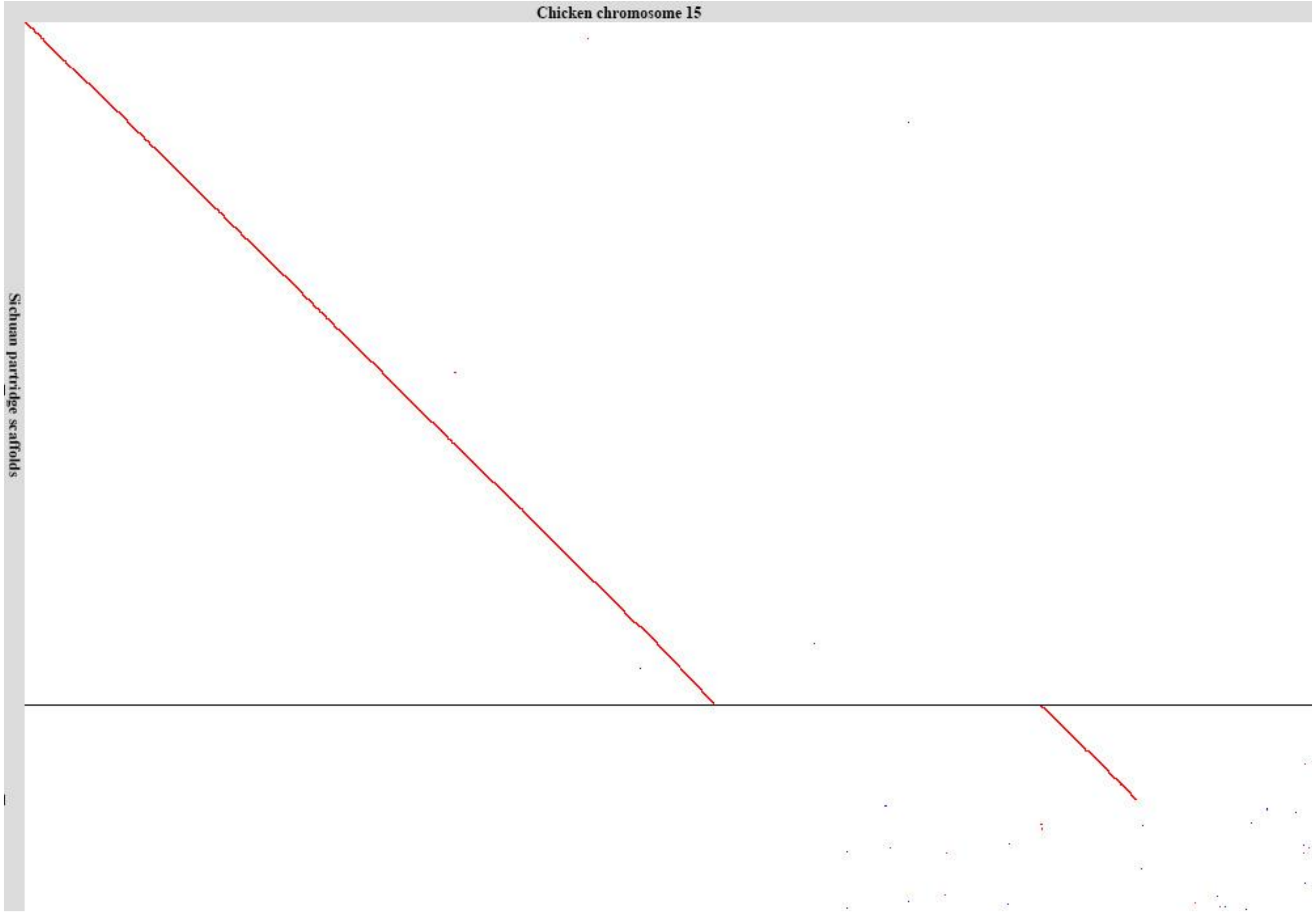

Chicken chromosome 15
Sichuan partridge scaffolds

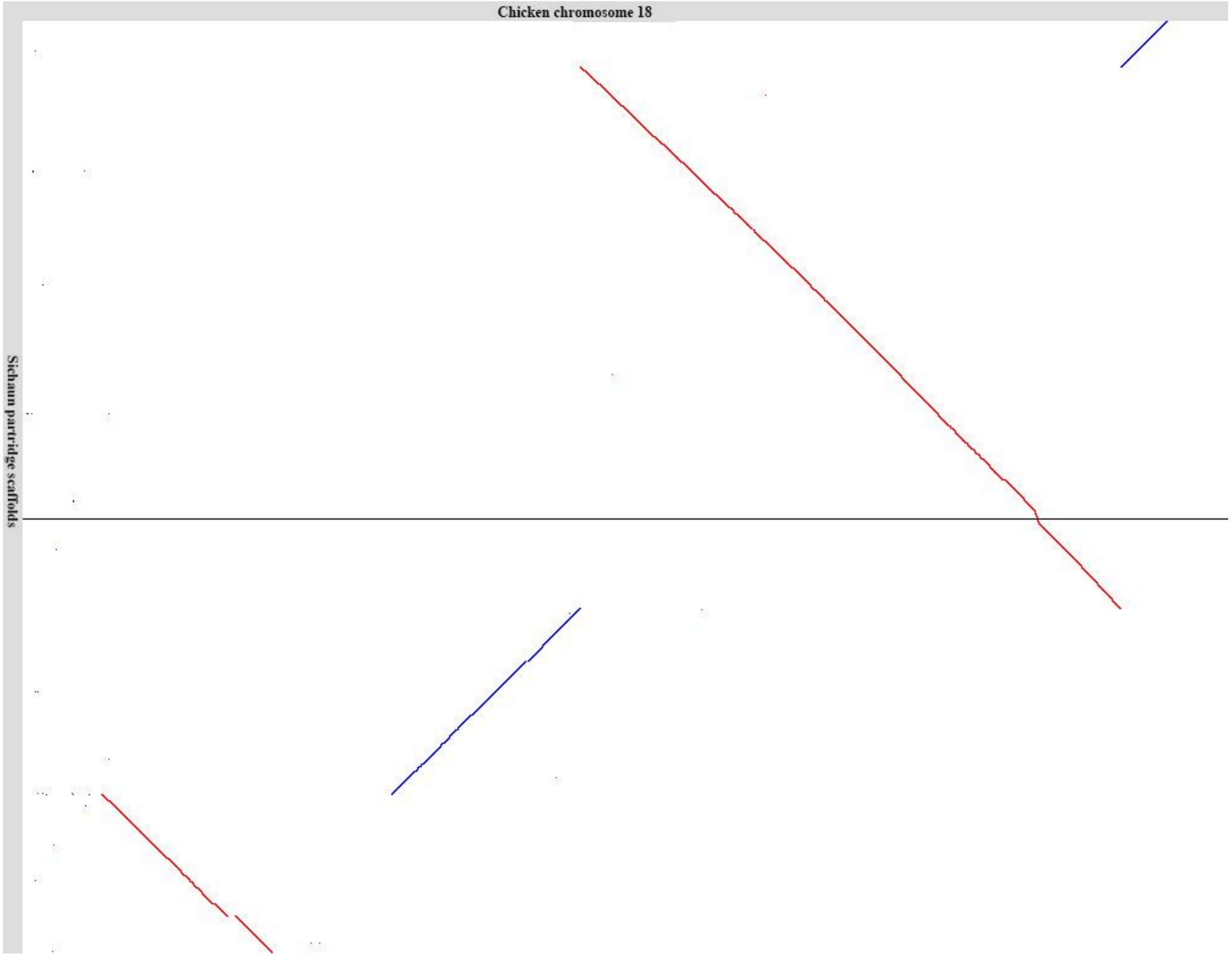
Chicken chromosome 18
Sichaun partridge scaffolds

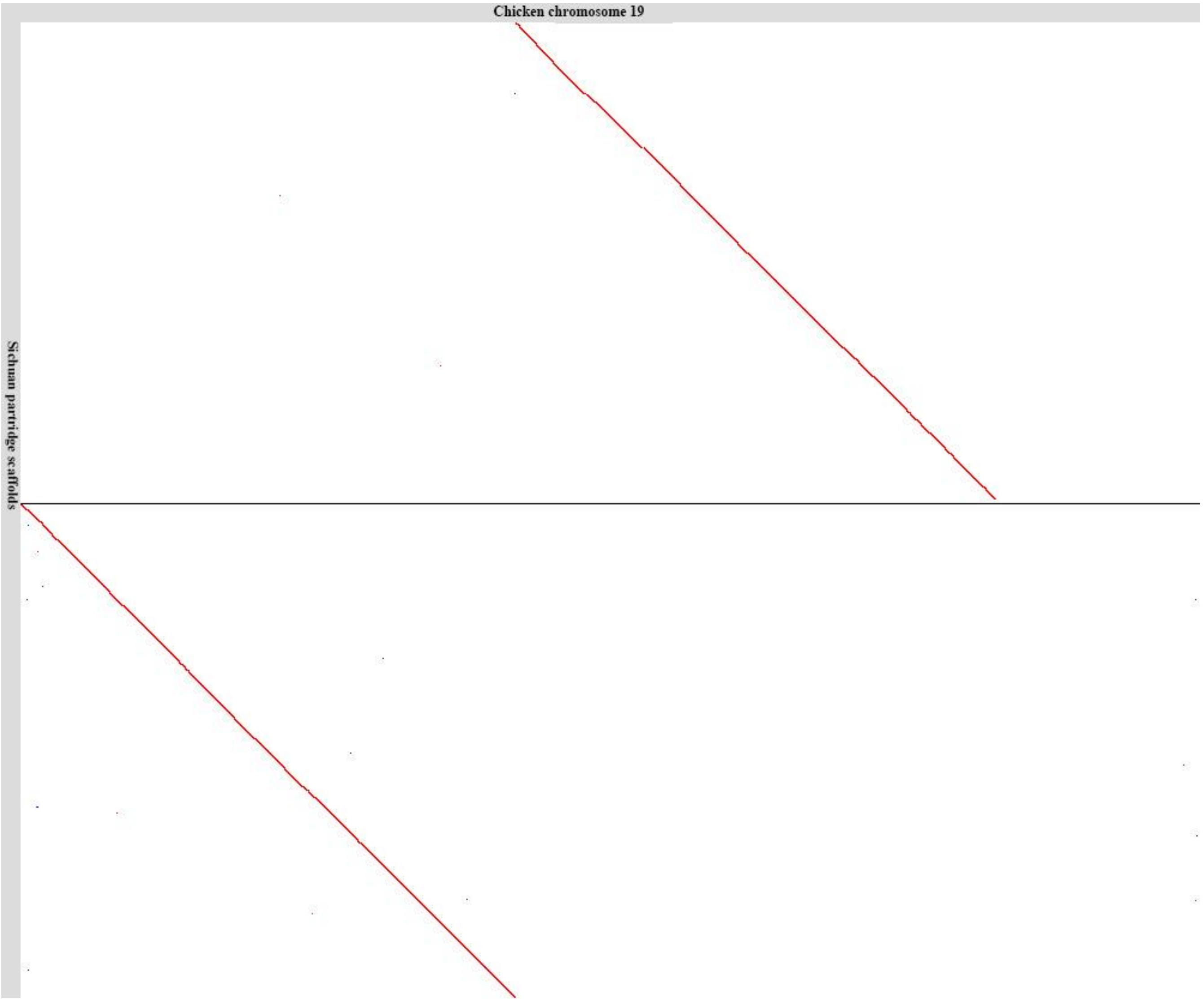
Chicken chromosome 19
Sichuan partridge scaffolds

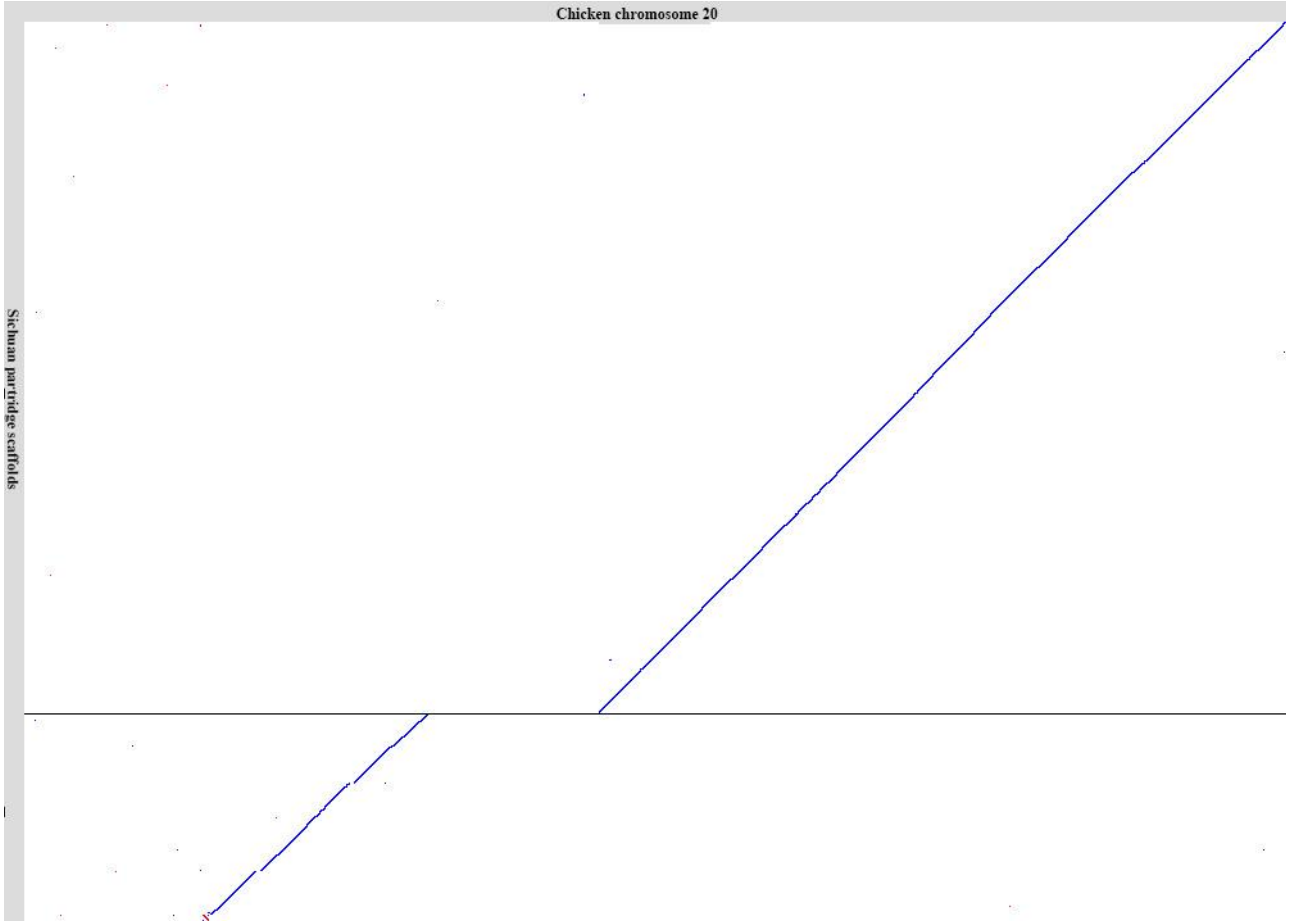
Chicken chromosome 20
Sichuan partridge scaffolds

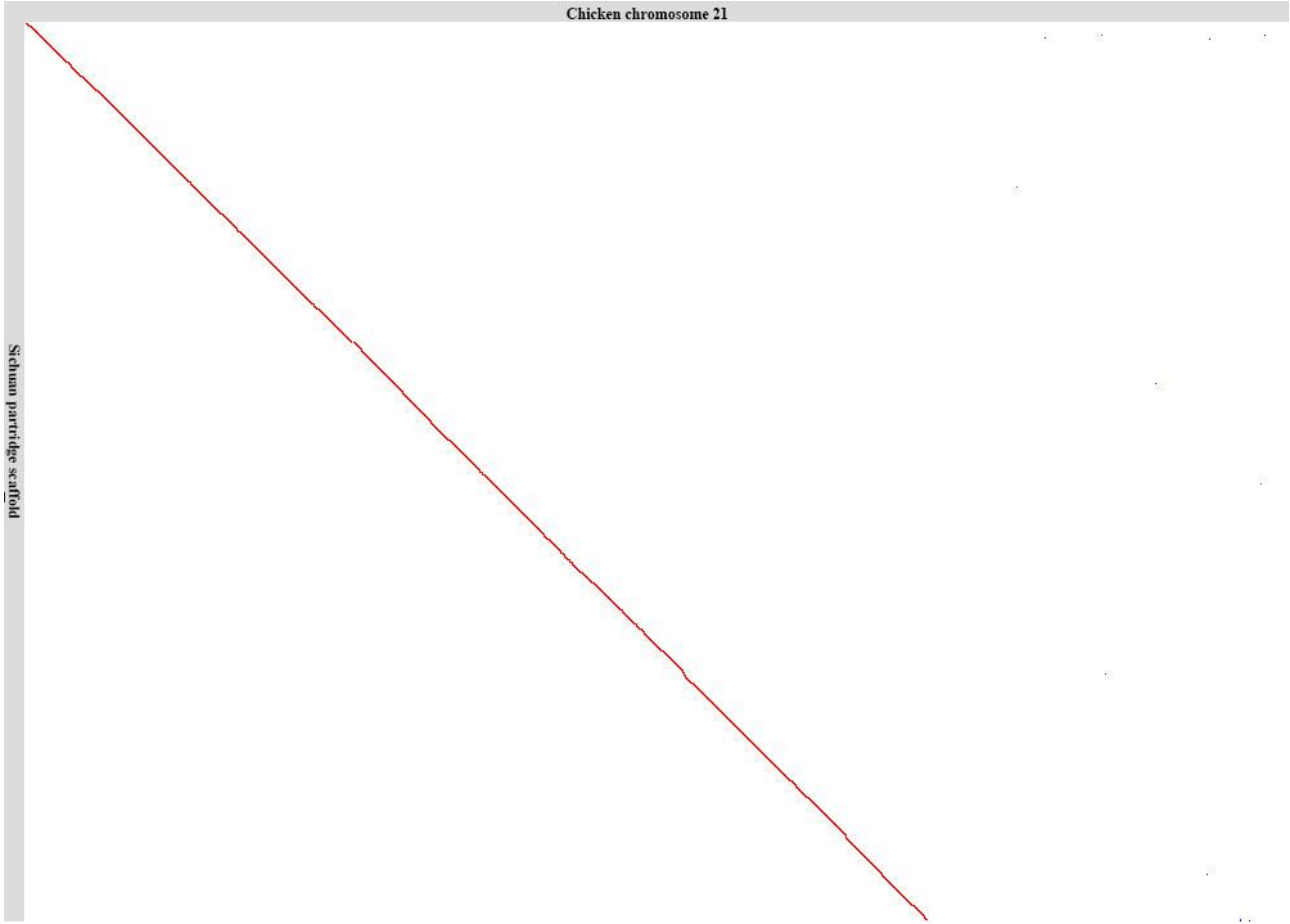
Chicken chromosome 21
Sichuan partridge scaffold

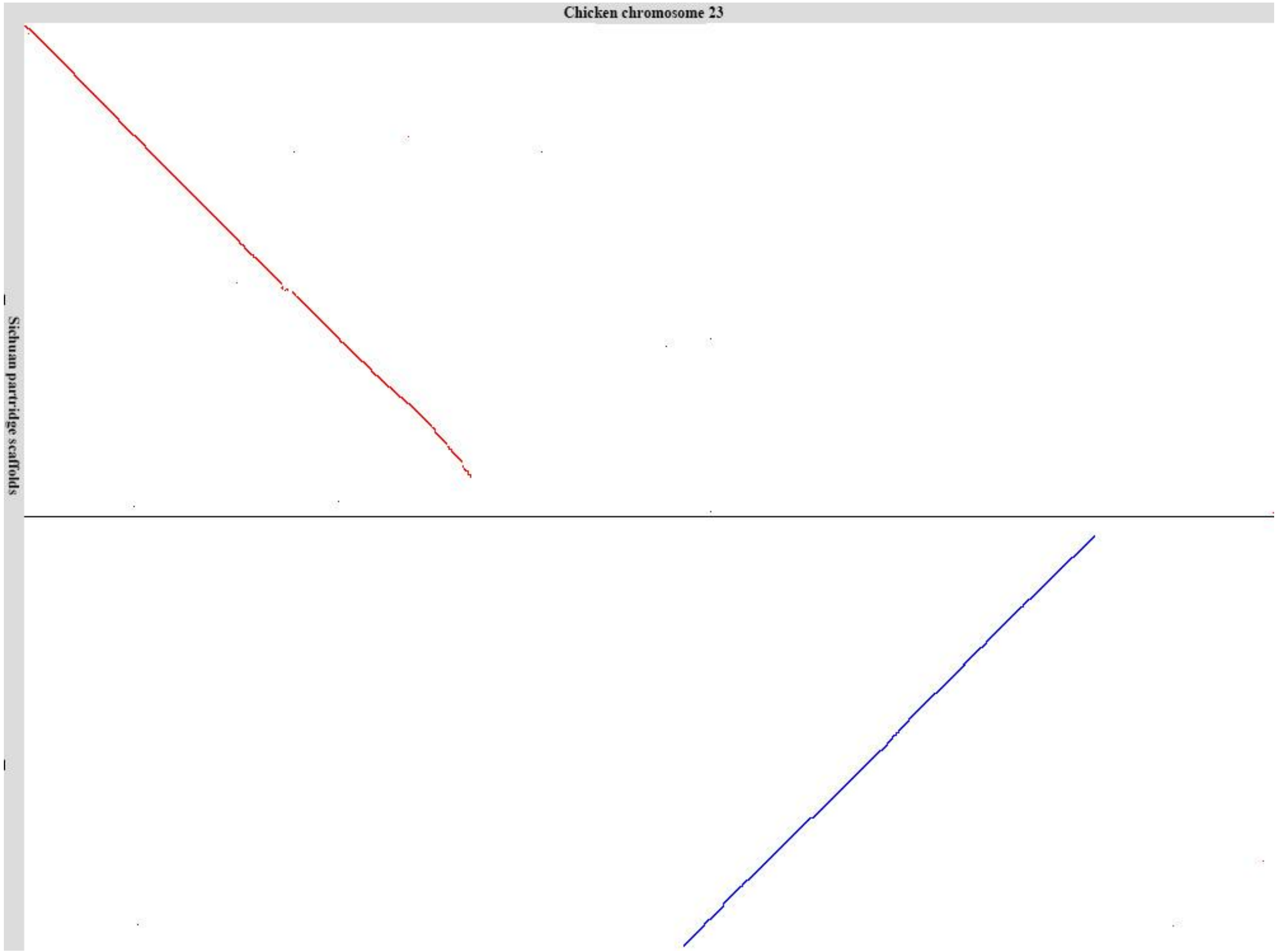
Chicken chromosome 23
Sichuan partridge scaffolds

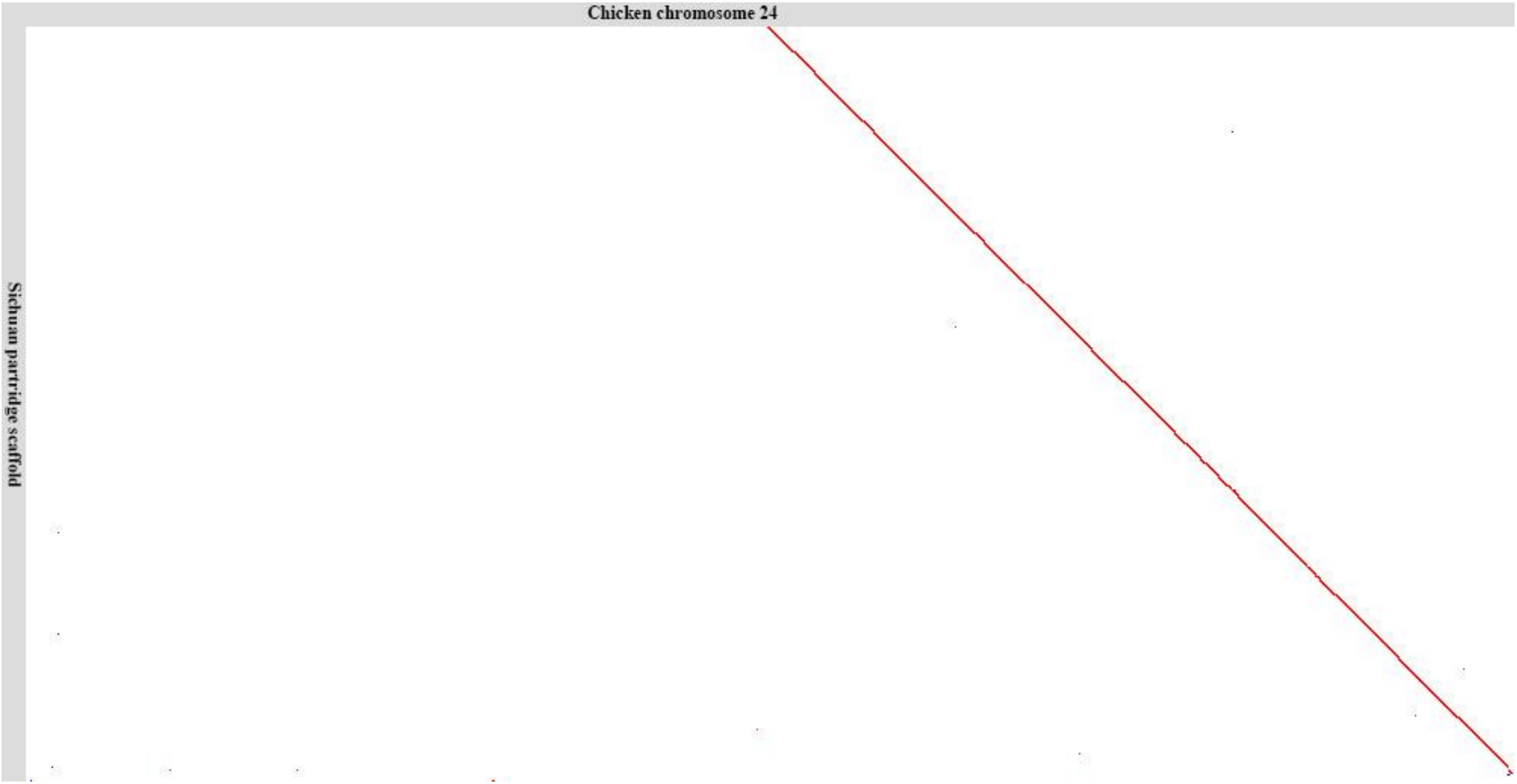
Chicken chromosome 24
Sichuan partridge scaffold

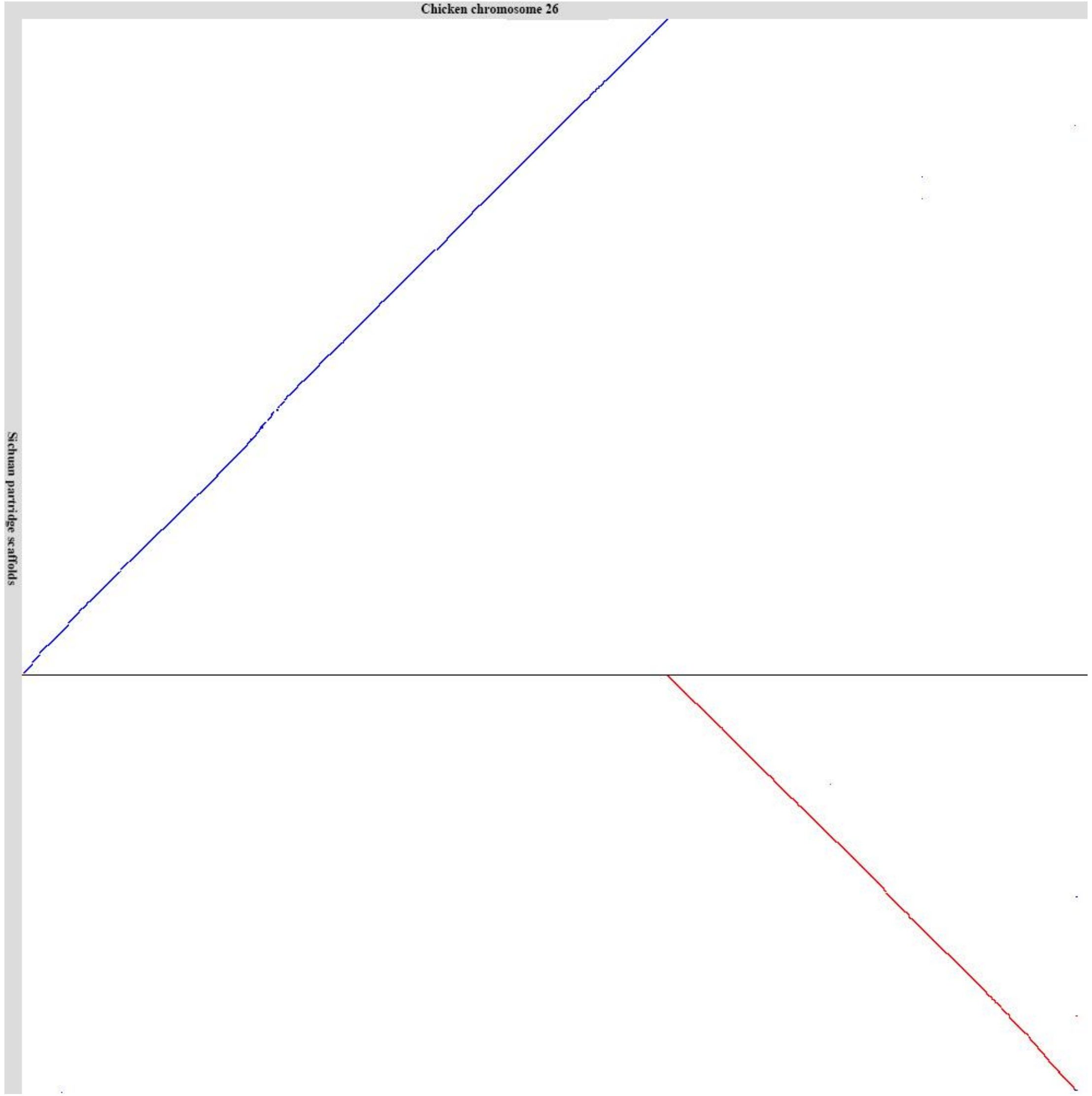
Chicken chromosome 26
Sichuan partridge scaffolds

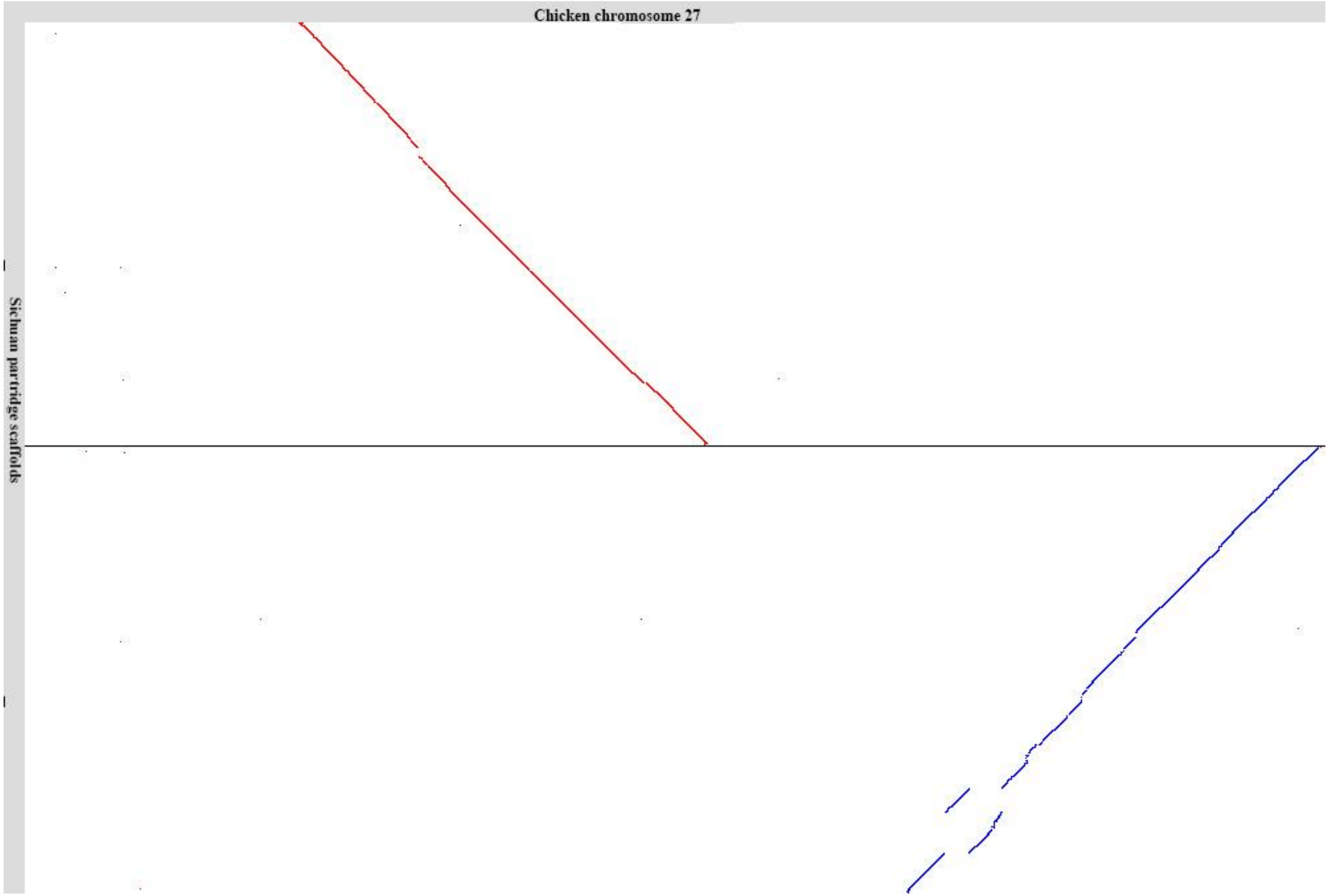
Chicken chromosome 27
Sichuan partridge scaffolds

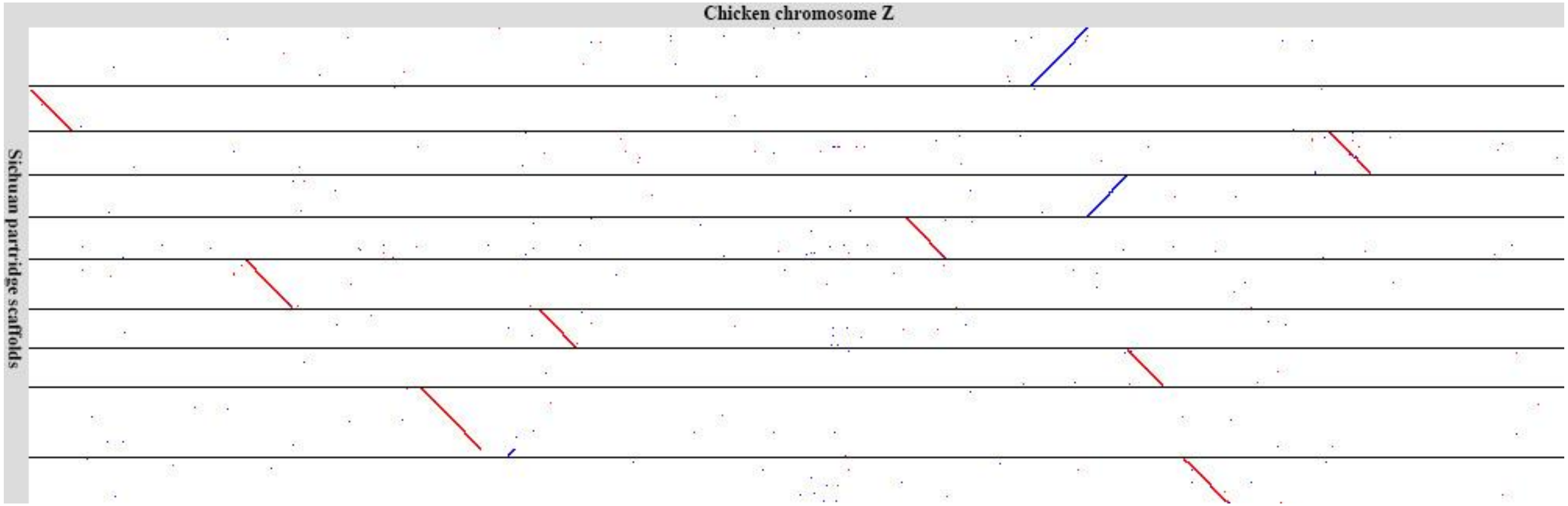

Chicken chromosome Z
Sichuan partridge scaffolds
